# Novel benthic foraminifera are abundant and diverse in an area of the abyssal equatorial Pacific licensed for polymetallic nodule exploration

**DOI:** 10.1038/srep45288

**Published:** 2017-04-06

**Authors:** Aurélie Goineau, Andrew J. Gooday

**Affiliations:** 1 National Oceanography Centre, University of Southampton Waterfront Campus, European Way, Southampton SO14 3ZH, United Kingdom

## Abstract

The benthic biota of the Clarion–Clipperton Zone (CCZ, abyssal eastern equatorial Pacific) is the focus of a major research effort linked to possible future mining of polymetallic nodules. Within the framework of ABYSSLINE, a biological baseline study conducted on behalf of Seabed Resources Development Ltd. in the UK-1 exploration contract area (eastern CCZ, ~4,080 m water depth), we analysed foraminifera (testate protists), including ‘live’ (Rose Bengal stained) and dead tests, in 5 cores (0–1 cm layer, >150-μm fraction) recovered during separate megacorer deployments inside a 30 by 30 km seafloor area. In both categories (live and dead) we distinguished between complete and fragmented specimens. The outstanding feature of these assemblages is the overwhelming predominance of monothalamids, a group often ignored in foraminiferal studies. These single-chambered foraminifera, which include agglutinated tubes, spheres and komokiaceans, represented 79% of 3,607 complete tests, 98% of 1,798 fragments and 76% of the 416 morphospecies (live and dead combined) in our samples. Only 3.1% of monothalamid species and 9.8% of all species in the UK-1 assemblages are scientifically described and many are rare (29% singletons). Our results emphasise how little is known about foraminifera in abyssal areas that may experience major impacts from future mining activities.

The Clarion–Clipperton Zone (CCZ), a vast tract of abyssal seafloor some 6 million km^2^ in area and 4,000–6,000 m deep in the eastern equatorial Pacific, hosts the World’s densest concentrations of polymetallic nodules. During the 1970s, the possibility of mining these deposits led to a major research effort in this region, aimed among other things at assessing the abundance, composition and diversity of the benthic biota that was at risk from any future mining activities^1^. Although some test mining was conducted, economic factors did not favour commercial operations at that time and interest in the benthic communities of the CCZ waned. During the past decade, however, as demand for metals such as nickel, copper, cobalt and rare earths has increased, partly as a result of developments in electronics, these potato-sized concretions have again become the focus of commercial interest, prompting renewed concerns about the mitigation of potential impacts from mining activities on seafloor biota^2^,^3^.

The CCZ lies outside national jurisdictions and the activities within it of companies and organisations are regulated by the International Seabed Authority (ISA), a body that was established under the United Nations Convention on the Law of the Sea. The ISA has issued a number of 15-year contracts for seafloor exploration in areas covering up to 75,000 km^2^ of seafloor. A major requirement of the ISA is that baseline studies of benthic biota should be carried out inside these contract areas before the next phase, the exploitation of the nodules, can be approved^4^. The UK-1 exploratory contract area at the eastern end of the CCZ is licensed to a company based in the United Kingdom, Seabed Resources Development Ltd., who are funding the ABYSSLINE (ABYSSal baseLINE) project in response to this requirement. ABYSSLINE is led by the University of Hawai’i at Mānoa and involves researchers from institutions in the USA, UK, Germany and Norway^5^. The present study is a contribution to this project and focuses on the foraminifera, a group of testate (shell-bearing) protists.

Foraminifera are an important component of benthic communities in many marine settings and particularly in the deep sea, where they are sometimes a dominant taxon in terms of both abundance and biomass. Certain components of these assemblages, notably calcareous and some multichambered agglutinated species, are well known from geologically-orientated studies^6^. In particular, research conducted during the Deep-Sea Drilling Project (DSDP) and its various successor programmes, most recently the International Ocean Discovery Program (IODP), have greatly increased our knowledge of the taxonomy of these ‘hard-shelled’ fossilisable taxa^7^. However, they typically represent a relatively small proportion of modern foraminifera in abyssal sediments. The dominant groups in such settings are often single-chambered monothalamous foraminifera (‘monothalamids’), many of them delicate, ‘soft-shelled’ forms^8^,^9^.

There are relatively few studies of abyssal Pacific foraminifera^6^. Cushman’s ‘Monograph of the Foraminifera of the North Pacific Ocean’^10^ combined new and literature records for 715 species, including ‘varieties’ (9.1% monothalamids). Later, Saidova’s 3-volume work ‘Benthic Foraminifera of the Pacific Ocean’^11^ included 1,796 species (10.05% monothalamids) from the entire Pacific (Supplementary Table S1). These massive studies were based on huge numbers of samples (>1,620 in the case of Saidova) spanning sublittoral to abyssal and hadal depth, mainly on the NE and NW margins of the Ocean with relatively few within the CCZ. In common with the vast majority of studies on deep-sea benthic foraminifera^6^, Cushman and Saidova both focused very largely on hard-shelled taxa. Similar studies covering smaller areas or specific sites in the North Pacific and based on fine sieve fractions (32–75-μm) have generally recorded <100 species. When delicate monothalamids, including komokiaceans, and fragmented tests are included^12^,^13^, species numbers are higher, well in excess of 100.

Here, we present the first comprehensive study of foraminifera in the >150-μm fraction of samples from the eastern CCZ (Table 1) and demonstrate that they are highly diverse and dominated by largely undescribed monothalamids. All of the studied samples were from an area that was rich in nodules. In order to facilitate comparison with previous studies we calculate diversity metrics that are often used in foraminiferal studies, as well as diversity estimates more commonly used in deep-sea biological surveys. Since monothalamous benthic foraminifera tend to be overlooked in foraminiferal studies, we performed additional statistical analyses and diversity estimations on two kinds of partial assemblages. 1) ‘Familiar’ species are those likely to remain intact in samples preserved in 95% ethanol and/or dried, i.e. using methods normal in micropalaeontological studies. They include all multichambered species (calcareous and agglutinated with an organic or calcareous cement), together with robust (but not delicate) monothalamids. 2) Fossilising species; following Mackensen *et al*.^14^, we include in this category all calcareous taxa in addition to *Eggerella bradyi*, the only agglutinated species in our material that uses calcareous cement.

## Results

In order to fully evaluate foraminiferal diversity, we included all specimens (live and dead, complete and fragmented) in our analyses. Species are recognised based on test morphology and structure, i.e. they are morphospecies. We have assigned these morphospecies to major groups, a mixture of formal taxa (e.g. rotaliids, hormosinids, komokiaceans) and informal morphology-based groupings (e.g. ‘tubes’, ‘spheres’). Based on their test characteristics, we regard species belonging to the Komokiacea (‘komoki’) as monothalamous foraminifera, although convincing molecular genetic data to confirm this placement are currently lacking. We also included gromiids, monothalamous testate protists related to foraminifera, which were encountered occasionally in our samples. Surface or subsurface nodules were present in some cores but were not present in the 0–1 cm layers of the five cores that were analysed.

### Abundance of complete and fragmented foraminifera

A total of 5,405 specimens was sorted from the top 1 cm layer of our 5 samples. Most (~94.5%) were assigned to species, in many cases undescribed (Table 2). The remaining specimens (398 complete and 113 fragments) were considered indeterminate, i.e. not sufficiently distinct to assign to a species; ~91% of these were monothamids. Two-thirds (3,607 = 66.7%) of all specimens were complete and the remainder (1,798 = 33.3%) were fragments. More than three-quarters (4,175 = 77.2%) were considered ‘live’ (i.e. Rose Bengal-stained), the remainder dead. Densities in individual samples ranged from 51.1 to 169.0 ind./10 cm^2^ for live complete specimens, and from 21.4 to 36.6 ind./10 cm^2^ for dead complete specimens (Supplementary Table S2). Fragment densities were lower: 21.8 to 97.1 live fragments/10 cm^2^ and 3.7 to 36.6 dead fragments/10 cm^2^.

### Overall assemblage composition

#### Multichambered foraminifera

Hormosinids were the most abundant multichambered group, representing 6.8% of all complete live specimens and 20.8% of complete dead specimens, and were virtually the only multichambered group represented by fragments (Table 2). Rotaliids accounted for 2.7% of all complete live specimens and 4.6% of all complete dead specimens. Three other calcareous groups, robertinids, lagenids and miliolids, were uncommon (<0.8% of complete live and dead specimens). Trochamminids and various other multichambered agglutinated foraminifera (i.e. ‘MAF’, excluding ammodiscids, hormosinids and trochamminids) were also important in the dead assemblage (6.0% and 11.8%, respectively), but were much less abundant in the live assemblage (0.4% and 1.1%, respectively).

#### Monothalamids

A heterogeneous assortment of tubular species (‘tubes’) formed the most abundant group of monothalamids in all categories, representing about one fifth of all complete specimens (21.9% of live, 19.3% of dead) and about a third to half of all fragments (36.7% of live, 48.5% of dead) (Table 2). Unclassified monothalamids, i.e. single-chambered individuals that could not be assigned to any formal taxon or morphology-based category, were the second-ranked group, making up 12.0% (live) and 4.9% (dead) of all complete specimens, and 25.1% (live) and 12.5% (dead) of all fragments. Another important group, the komokiaceans (particularly members of the family Baculellidae), made up 21.3% (live) and 20.1% (dead) of all fragments, and were also fairly common in the live complete assemblage (7.6%). A group combining the genus *Lagenammina* and morphologically similar flask-like species accounted for 7.6% (live) and 15.3% (dead) of all complete specimens. Spherical species (‘spheres’ including psammosphaerids) made up 23.6% (live) and 2.8% (dead) of all complete specimens.

Many of the monothalamids were fragmented, although the proportion varied between groups. Almost half (47.8%) of tubular and more than half (52.0%) of unclassified specimens were fragments. The proportion was even higher in the case of chain-like foraminifera (77.6%) and komokiaceans (63.4%). On the other hand, all spheres and members of the *Lagenammina*/flask group were considered to be complete.

### Species-level composition

#### General characteristics

Based on test morphology, we recognised a total of 416 species, of which 317 were monothalamids, 96 were multichambered, and 3 were gromiids (Table 3). Live complete specimens yielded the largest number of species (355), followed by dead complete specimens (131), live fragments (84), and dead fragments (43). The number of species confined to one of these categories, i.e. the number added if the category is included (‘S_add_’ in Table 3), followed the same trend: live complete 222, dead complete 36, live fragments 12 and dead fragments 2 species.

Rare species were frequent (Supplementary Figure S1 and ‘Singl.’ in Table 3); 276 species were represented by 5 or fewer complete specimens, and 122 (29.3%) were singletons, of which the majority (90 = 73.7%) were monothalamids. Many taxa are undescribed; 113 (27.6%) were identified to a known genus (but not to a known species), and only 41 (9.8%) to a described species. The numbers of multichambered (54) and monothalamous (59) taxa assigned to genus (but not species) were almost equal, whereas the majority of described species (31) were multichambered.

There were clear differences between the monothalamid and multichambered groups in the proportions of live and dead species (Table 3). In total, we recognised 305 live monothalamid species (complete specimens and fragments combined), compared to only 86 species represented by dead specimens. Monothalamids dominated the live complete assemblage (282 species = 79.4%) and accounted for almost all species occurring as live (83 species = 98.8%) and dead (38 species = 88.4%) fragments. The dominance of live compared to dead monothalamid species contrasted with the equal numbers of multichambered species that were judged to be either live (70) or dead (69). Although, in total, there were more than three times as many monothalamous than multichambered species (317 vs 96), slightly more multichambered (69) than monothalamous (62) species included at least some dead specimens, while the number of multichambered species represented only by dead specimens was more than double that of monothalamids (26 vs 10; S_add_ in Table 3). All the species occurring only as fragments (21 live, 11 dead) were monothalamids.

#### Multichambered foraminifera

The most specious multichambered groups were the hormosinids (29 species), various MAF (22), and trochamminids (12), all of them agglutinated (Table 3). Only 30 species (live and dead) were calcareous; of these, 19 were rotaliids, 5 were lagenids, 5 were miliolids and 1 was a robertinid. Most hormosinids (28 out of 29) and rotaliids (17 out of 19) were represented by complete live specimens, whereas most trochamminids (10 out of 12) and other various MAF (21 out of 22) were represented by complete dead specimens. Only 6 [Fig f1][Fig f2]multichambered species (5 hormosinids and 1 MAF) were found as fragments.

#### Monothalamids

Spheres (66 species), tubes (65), komokiaceans (43) and unclassified monothalamids (42) were the most specious monothalamid groups (Table 3). The vast majority of the spheres (64 out of 66) and unclassified monothalamids (38 out of 42) were represented almost entirely by live complete specimens. Most tubular (52 out of 65) and komokiacean (34 out of 43) species occurred as complete live specimens, but they were also the most specious groups among fragments, live (39 and 20 species, respectively) as well as dead (18 and 8 species, respectively). Among the 59 monothalamid species identified to a known genus (but undescribed species), 19 were komokiaceans and 10 were tubular species. Almost half (30 out of 66) of the spherical species were singletons.

### Top-ranked species

The majority (21) of the thirty most abundant species occurring as [Fig f3]complete specimens (live and dead combined) were monothalamids (Figs 4 and 5, Supplementary Table S4, Supplementary Material S1, and Supplementary Figures S2–S11). They included tubes (7 species), unclassified monothalamids (4), *Lagenammina* plus flasks (3), spheres (2), komokiaceans, family Komokiidae (2), komokiacean-like (1), *Nodellum*-like (1) and chain-like (1) forms. Only 9 multichambered species (5 hormosinids, 1 ammodiscid, 1 rotaliid, and 2 MAF) were included in the top 30. Among the top-10 species, 8 were monothalamids while only two (*Nuttallides umbonifera, Reophax scorpiurus*) were multichambered. Although the majority of the top-30 species occurred in most samples (i.e. 27 species in at least 3 samples), only 4 species were among the top 10 in 3–4 samples, and none were in the top 10 of all samples.

The 30 most abundant species occurring as fragments (live and dead combined) were monothalamids, with a mixture of tubes (17), komokiaceans, families Baculellidae (3) and Komokiidae (3), chain-like (4), unclassified (3) and komokiacean-like (1) species. Seven of these species also occurred in the top-30 ranked species based on complete specimens. Only 3 of the top-10 species (*Edgertonia* sp.4 and sp.7, *Rhizammina* sp.1) could be identified to a known genus.

### Species richness, Alpha diversity and estimated diversity

**Figure 1 f1:**
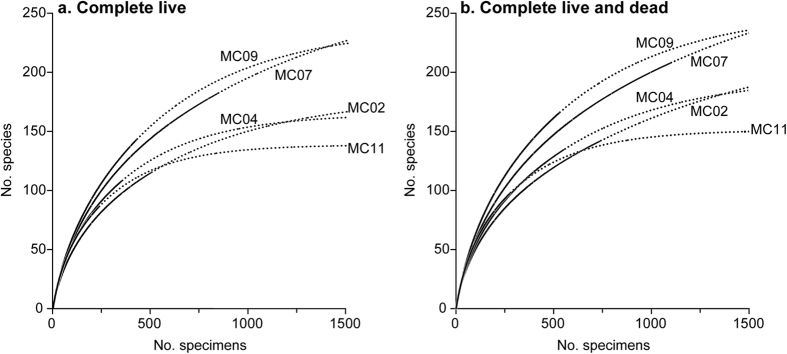
Rarefaction curves based on complete live (**a**) and complete live and dead (**b**) specimens. Solid black lines are based on actual data; dashed lines are extrapolated curves up to 1,500 specimens for each sample.

**Figure 2 f2:**
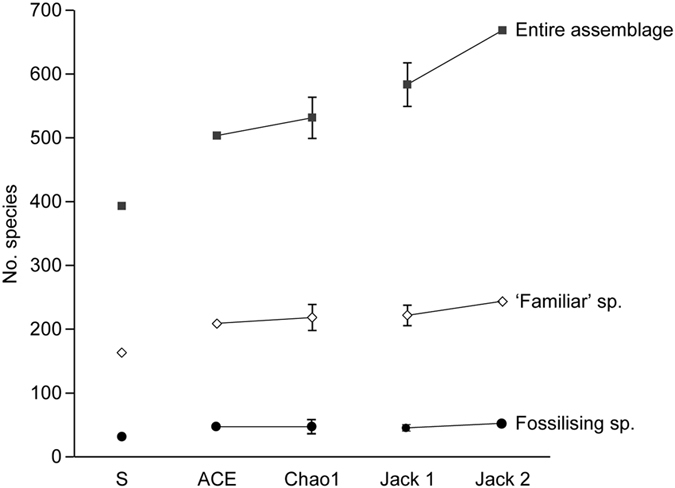
Total numbers of species recognised in the 5 samples (S) and estimated number of species for the entire assemblage and for partial assemblages (fossilising species and ‘familiar’ species). Abundance-based estimators ACE and Chao1 were calculated based on complete specimens, live and dead combined; incidence-based estimators Jacknife 1 and 2 (‘Jack 1’, ‘Jack 2’) were calculated based on complete specimens and fragments, live and dead combined.

The entire assemblage in each sample yielded 86 to 182 species represented by complete live tests, corresponding to 24.2–51.3% of the total number of complete live species (355) recognised among all complete live specimens (Table 4). Shannon (H) and Fisher alpha (α) indices indicated a very high diversity, with values of 3.93–4.42 and 46.4–74.8 per sample, respectively. Evenness index (E) was also high (0.44 to 0.69). Rarefaction curves did not reach an asymptote for any of our 5 samples (Fig. 1a). The extrapolation of these rarefaction curves tended to level off for MC04, MC09 and MC11 at 750–1,500 individuals, whereas the extrapolated number of species was still increasing at >1,500 individuals in the case of MC02 and MC07. Chao1 estimated the actual diversity of our individual samples between 134 ± 20 and 273 ± 28 species. The addition of complete dead specimens to the complete live database increased all diversity indices and diversity estimates (Table 4), although the corresponding rarefaction curves followed the same trend as those based only on complete live specimens, i.e. they did not level off (Fig. 1b). The Chao1 values for each individual sample ranged from 147 ± 20 to 293 ± 25 species, an increase of ~10–30% compared to the complete live assemblage. When combining our 5 samples, abundance-based estimators (i.e. based on the number of complete specimens, live and dead combined) ACE and Chao1 estimated a total foraminiferal diversity of 503 and 531 ± 32 species, respectively. The incidence-based estimators (i.e. based on the occurrence of complete specimens and fragments, live and dead combined) Jacknife 1 and 2 estimated totals of 583 ± 34 and 668 species, respectively (Fig. 2).

When partial assemblages, i.e. ‘familiar’ and fossilising species, were considered, diversity indices and estimates decreased substantially (Table 4). Based on complete live and dead specimens, Chao1 for each individual sample ranged from 58 ± 12 to 155 ± 20 for ‘familiar’ species, and from 1 ± 0 to 29 ± 11 for fossilising species, estimates that are 48–62% and 88–99% lower, respectively, than for the entire assemblage. The estimated total diversity of these partial assemblages for our 5 samples ranged from 209 to 243 (‘familiar’ species) and 45 to 51 (fossilising species), i.e. only ~31–48% and ~7–10%, respectively, of the estimated diversity based on the entire assemblage (Fig. 2).

## Discussion

The research reported here represents the most complete available analysis of foraminiferal diversity in larger size fractions (>150-μm) of abyssal Pacific sediments. It demonstrates that foraminifera in undisturbed core-top samples are extremely diverse, include many rare species, are dominated by monothalamids that are often delicate and prone to fragmentation, and include a large proportion of species that are unknown to science (including >95% of the monothalamids). Most faunal data on abyssal foraminifera concern only the multichambered taxa and the more robust monothalamids (our ‘familiar’ assemblage). The few studies that include the delicate ‘less familiar’ monothalamids (our ‘entire’ assemblage) have been carried out mainly in the Atlantic^15^,^16^, Indian^17^ and Southern^18^ Oceans. In the central North Pacific (5,599–6,036 m depth), Bernstein *et al*.^12^ counted complete (‘unit’) and fragmentary specimens and species (including komokiaceans) in the >297-μm fraction of 5 box cores, but did not identify species beyond genus level. A subsequent taxonomic re-evaluation of this material by Schröder *et al*.^19^ considered only part of the collection. Most other studies in the CCZ have likewise addressed selected components of the foraminiferal faunas^20^,^21^,^22^,^23^,^24^. Our study of ‘entire’ assemblages is closest to that of Nozawa *et al*.^13^ who analysed samples from the central CCZ. Their study complements ours by being based on much finer (32-μm) fractions of much smaller (3.45 cm^2^) samples.

### The dominance of little-known monothalamids

In existing Pacific deep-sea benthic foraminiferal datasets, the most specious groups are generally the rotaliids and multichambered agglutinated taxa with monothalamids being a relatively minor component (Supplementary Table S1). In striking contrast, our study reveals that, in terms of both specimens and species, foraminiferal assemblages in the UK-1 Stratum A are dominated by monothalamous taxa, notably tubular and spherical species, komokiaceans and various unclassified species. Hormosinids (*Reophax* and its allies) constituted the most abundant and diverse group of multichambered taxa, while calcareous taxa (mainly rotaliids) were rare, perhaps related to the proximity of the carbonate compensation depth. These results are consistent with studies conducted at abyssal sites in the North Atlantic^15^,^25^, Indian^17^, Southern^18^ and Pacific^13^,^26^ Oceans which have revealed that monothalamous foraminiferans often constitute >60% of the foraminiferal assemblages, with maximal contributions (>87%) recorded at relatively oligotrophic sites on the Cape Verde Abyssal Plain^15^ and in the eastern equatorial Pacific (Kaplan East site)^13^. The higher proportion of monothalamids in the abyss may be related to a reduced dependence, compared with multichambered and particularly calcareous taxa, on fresh organic matter (OM) derived from surface phytoplankton production^27^. This idea is supported by feeding experiments at Station M (NE Pacific), where ‘soft-shelled saccamminids’ showed almost no uptake of labelled carbon compared to other agglutinated and calcareous foraminifera^28^. The accumulation of stercomata by many monothalamids in oligotrophic abyssal settings suggests that they are deposit feeders ingesting sediment, associated bacteria and perhaps low-quality OM^8^. Tendal^29^ suggested that stercomata could be used as ‘bacteria farms’, the host subsequently feeding on the grown bacteria, but no direct evidence exists for this hypothesis. Symbiotic associations between prokaryotes and benthic foraminifera have been observed in several calcareous species, from shallow tropical^30^,^31^ to bathyal environments^32^. Such associations are possibly common in deep-sea foraminifera, particularly in oligotrophic areas like the CCZ. In addition, the delicate stercome-bearing foraminifera that are typical of these deep-sea oligotrophic environments often have complex morphologies, which likely limit their movement, if any, within the sediment to levels well below the few tens of microns per minute recorded for some shallow-water species under laboratory conditions^33^,^34^. This and other indirect lines of evidence^8^ suggest that monothalamids have slower growth and metabolic rates compared to the multichambered taxa that are often highly active in terms of carbon processing^35^,^36^,^37^. Nevertheless, gut content analyses and other evidence of feeding by deep-sea macrofauna and megafauna^38^,^39^ suggest that incidental and targeted consumption of komokiaceans, xenophyophores (giant foraminifera confined to the deep-sea), and probably other stercomata-bearing monothalamids, may be widespread in the abyss. The high abundance of monothalamids in our samples and elsewhere in the Pacific make it likely that they play a substantial role in carbon cycling over vast areas of the Pacific Ocean floor by consuming refractory OM and making it available to higher trophic levels.

### Very high foraminiferal diversity

Our analyses of foraminifera in the >150-μm fraction of 5 cores (0–1 cm layer) yielded 416 species, with between 116 (493 specimens) and 237 (1,949 specimens) being identified in individual samples. Different methods make comparisons difficult. However, it is notable that this figure is almost a quarter of the total number of species (1,796) recorded by Saidova^11^ from >1,630 stations spanning the entire Pacific Ocean, more than twice the 164 species recorded by Burke^40^ from 29 samples spanning 2,721 m of water depth on the Ontong Java Plateau, several times more than the 117 species recorded by Enge *et al*.^28^,^36^ in >63 μm fractions from Station M, and an order of magnitude more than the 55 benthic species recorded by Smith^41^ in the >75-μm fraction from 27 abyssal sites in the North Pacific (Supplementary Table S1). The number of species in our samples also substantially exceeds the 141 recovered from 5 box core subcores (>297-μm fraction) in the North Pacific^12^ and the 252 from syringe subsamples of megacorer cores (>32-μm fraction) from the eastern CCZ^13^ despite the inclusion of delicate monothalamids in these earlier studies. Overall, species numbers and diversity metrics in individual samples are somewhat higher than those reported from abyssal sites in the NE Atlantic, where delicate monothalamids were also analysed^15^,^25^,^42^. Comparisons, however, are compromised by the exclusion of dead tests and the analysis of finer (>63 μm) size fractions in the Atlantic studies.

The UK-1 species comprised a few common and numerous rare species, many of them undescribed. This pattern is typical for deep-sea foraminiferal assemblages from well-oxygenated settings^43^. Metazoans exhibit similar assemblage characteristics. In the abyssal Angola Basin (SE Atlantic), 5 megacorer deployments yielded 682 species of harpacticoid copepods, of which 56% were singletons and 99.3% were new to science^44^. A total of 223 box cores from 10 stations on a 176-km transect along the 2,100-m depth contour in the NW Atlantic yielded 798 macrofaunal species, 21% of them singletons, 11% doubletons and 460 undescribed^45^. Molecular studies of macrofauna diversity across the CCZ (French and German contract areas) have revealed similarly high numbers of Molecular Operational Taxonomic Units (MOTUs)^46^.

The phenomenon of high species diversity (e.g. high niche partitioning) on the food-poor abyssal seafloor is still not fully understood^47^,^48^. One popular idea invokes the patch dynamics^49^ whereby seafloor heterogeneity arising from patchy food inputs and physical disturbances supports assemblages of species at different successional stages within a relatively small area^47^. We have previously suggested that radiolarian tests represent an important source of habitat heterogeneity for small foraminifera at sub-centimetre scales in the UK-1 area and presumably other parts of the equatorial Pacific^20^. The activities of metazoans will create additional heterogeneity^47^. Polymetallic nodules are, at least visually, the most obvious source of seafloor heterogeneity within UK-1 Stratum A^50^. In the CCZ, nodule fields yielded more diverse assemblages of nematodes^51^,^52^ and megafauna^50^,^53^ than nodule-free areas. There were no nodules in the surficial layers of the five cores that we analysed and it is unclear whether foraminiferal diversity is influenced by the mere proximity of nodules. A more important factor may be that many monothalamids have limited mobility, a likely consequence of their complex test morphologies. This would allow them to occupy specialised ecological niches, leading to the co-existence of many species in heterogeneous, nodule-rich habitats. On the other hand, the nodules themselves host rich assemblages of encrusting foraminifera, the majority of which are not represented in the sediment community^21^. Their presence, therefore, will undoubtedly enhance the diversity of the foraminiferal assemblages as a whole (i.e. sediment- plus nodule-dwelling species) within our study area and across the CCZ.

### The reliability of diversity estimates

**Figure 3 f3:**
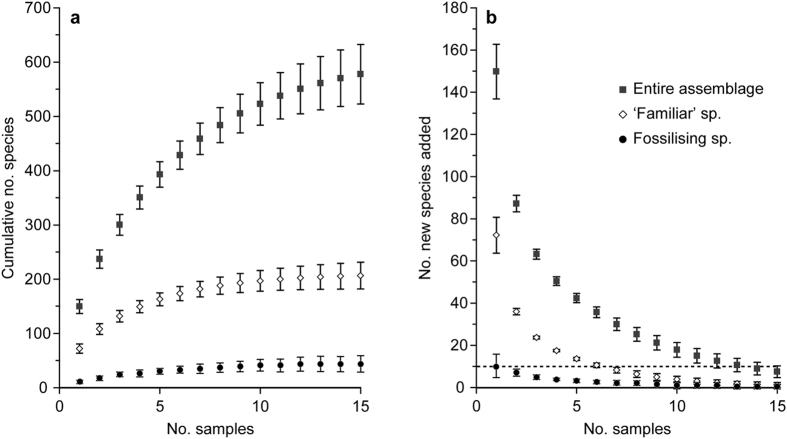
Sample-based rarefaction curves based on the number of complete (live and dead combined) specimens from the 5 analysed samples combined, and extrapolated up to 15 samples (**a**). Corresponding number of new species added per additional sample based on the sample-based extrapolated rarefaction curves (**b**). Estimations are shown for the entire assemblage and for partial assemblages (fossilising species and ‘familiar’ species). For each sample, vertical bars indicate the 95% interval confidence.

**Figure 4:**
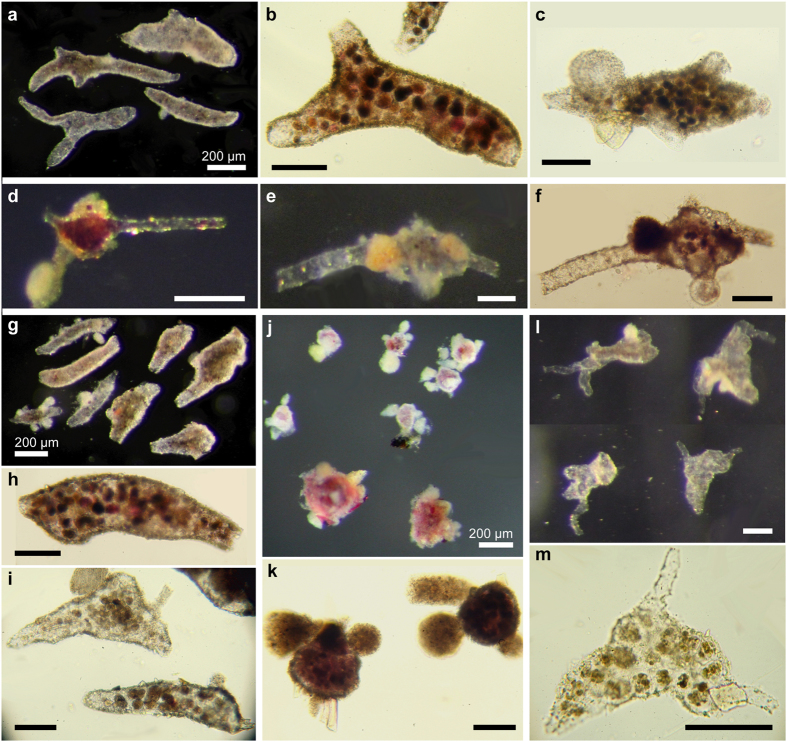
Reflected and transmitted light photographs of the 5 most abundant species represented by complete specimens (live and dead combined). (**a**–**c**) Tube sp. 48. (**d**–**f**) Flask sp. 4. (**g**–**i**) Tube sp. 54. (**j**–**k**) Psammosphaerid sp. 19. (**l**–**m**) Komokiacean-like sp. 20. All scale bars 100 μm except where indicated otherwise.

**Figure 5:**
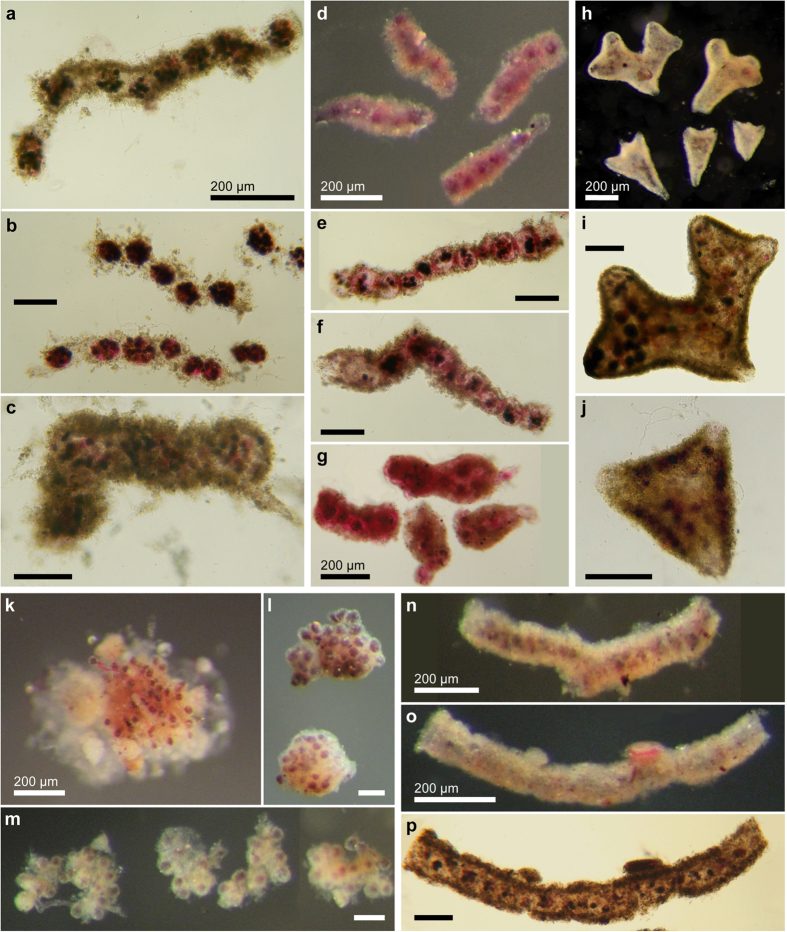
Reflected and transmitted light photographs of the 5 most abundant species represented by fragmented specimens (live and dead combined). (**a**–**c**) Monothalamid sp. 32. (**d**–**g**) Chain sp. 13. (**h**–**j**) Monothalamid sp. 27. (**k**–**m**) *Edgertonia* sp. 7; specimens k and l were considered to be complete. (**n**–**p**) Tube sp. 43. All scale bars 100 μm except where indicated otherwise.

Individual samples were very diverse with >100 species in the >150-μm fraction of single cores (0–1 cm). The number of species recognised in each individual sample corresponds to 62–71% of the number of foraminiferal species estimated by the Chao1. In an environmental DNA study in the Southern Ocean, between 51% and 82% (average 71%) of the total number of MOTUs in particular multicorer deployments were found in single cores from those deployments^54^. By analogy with Lejzerowicz *et al*., we regard our Chao1 values from single samples as estimates of the number of foraminiferal species in this size fraction and sediment layer that would be expected to occur in all 12 cores from the same megacorer deployment as the analysed core. Similarly, diversity estimates for all samples combined (503–668 species) are assumed to represent the total number of foraminiferal species that would be expected in the >150-μm fraction, 0–1 cm layer of all 60 cores from all 5 deployments. The actual combined number of species (416) recognised in our 5 samples represents 62–83% of these estimated total number of species. Sample-based rarefaction curve, extrapolated up to 15 samples, based on complete live and dead specimens (entire assemblage), tended to level off after 12 samples (Fig. 3a), each additional sample yielding less than 10 new species (Fig. 3b). This suggests that a total of 15 samples will yield the majority of species in this fraction of the top layer. For the ‘familiar’ and fossilising assemblages, curves tended to an asymptote after 6 and 8 samples, respectively (Fig. 3a).

Although our samples yielded numerous foraminiferal species, the number living in the UK-1 Stratum A is certainly much higher. Shipboard sorting of unfixed megacorer sediments (>150-μm fraction) and epibenthic sledge residues (>300-μm fraction) for molecular studies during the AB01 cruise yielded species that did not occur in any of our 5 samples. More importantly, we will have missed many species by analysing only the 0–1 cm layer and the >150-μm fraction. It is well documented that foraminifera live in the deeper layers of deep-sea sediments, and in some cases are confined to them^9^,^43^,^55^,^56^,^57^. Sieve residues between 63 and 150 μm are very productive in terms of species^25^,^43^, small opportunistic species often being confined to this fraction^58^. The 32–63-μm^13^,^59^ and <32-μm^56^,^60^ fractions yield additional tiny species^59^,^61^. The nodules themselves are colonised by rich assemblages of largely undescribed foraminiferal species, either encrusting the outer surface of the nodules^21^,^24^,^62^ or their inner cavities and crevices^63^, with very little overlap between the nodule- and sediment-dwelling assemblages. If these additional sources of diversity are taken into account, our estimate of 503–668 foraminiferal species, based on only 5 samples, is certainly a considerable under-estimate of the number of foraminiferal species across the entire UK-1 Stratum A area.

### Outstanding questions and future directions.

(1) Analysis of monothalamids can be very laborious; in the present study, the analysis of each core involved several months of work. The more robust multichambered taxa, the main focus of most foraminiferal studies, are easier to sort and identify. These more familiar taxa have been used to monitor the impact of anthropogenic activities on the marine environment since the late 1950’s^64^,^65^. Over recent decades, the number of such studies has substantially increased from shallow^66^ to deep-sea^67^ environments. This led to the FOBIMO (Foraminiferal Biomonitoring) initiative, developed by a group of micropaleontologists to standardise methods by making a series of recommendations. One ‘mandatory’ recommendation is that ‘*soft*-*shelled species* (which includes many monothalamids) *are not considered in routine monitoring studies*’^68^. This may be defensible for monitoring studies that rely on indicator species, but for baseline biodiversity assessment, it is essential to include monothalamids. In the present study, analysis of ‘familiar’ or fossilising species would have missed up to ~70% and 93%, respectively, of the foraminiferal diversity.

(2) Important insights into the diversity of deep-sea benthic foraminifera have been obtained from metabarcoding analyses of environmental DNA samples (small volumes of surficial sediment) using High Throughput Sequencing (HTS) technologies. These have revealed numerous MOTUs dominated by a mixture of monothalamids and foraminiferal barcodes that cannot be assigned to any known higher taxonomic groups^54^,^69^, many of the latter probably being artefacts^70^. In a general sense, the high diversity of foraminifera revealed by these genetic studies, and the relative proportions of MOTUs assigned to different major groups, are consistent with the morphology-based results reported here. However, the majority of deep-sea foraminiferal metabarcodes cannot be assigned to known species while the formalin-fixed material on which the present study is based is unsuitable for DNA analysis. In addition, the highly diverse monothalamids, particularly stercomata-bearing forms such as komokiaceans, isolated individually from unfixed deep-sea samples often fail to yield convincing sequences^71^. The integration of deep-sea foraminiferal diversity datasets based on molecular and morphological data therefore represents a major challenge.

(3) The standardisation of taxonomy across the CCZ is an important requirement of the ISA, a central objective of the ABYSSLINE project, and essential for the establishment of biogeographic patterns^72^. Morphology-based comparisons^6^,^7^,^19^,^27^ and molecular genetic data^69^,^73^ suggest that many deep-sea foraminifera, including monothalamids, have wide geographical distributions at abyssal depths. This is consistent with the hypothesis that free-living eukaryotes smaller than 1 mm occur worldwide wherever their required habitats are realised (‘everything is everywhere’)^74^. Yet abyssal sediments typically contain species that have not been recorded elsewhere^75^, an observation supported by environmental DNA studies^69^. These contrasting observations underline our currently poor understanding of deep-sea foraminiferal biogeography. Improved knowledge will be the key to estimating the potential vulnerability and probability of extinction of these abyssal protists resulting from future deep-sea mining activities in the CCZ.

(4) Improved biogeographic knowledge is also essential for estimating the number of foraminiferal species at larger spatial scales. Rarefaction curves indicate that we did not sample all sediment-dwelling species in the >150-μm fraction, and the number (416) of species recognised would have been higher if finer sieve fractions (63–150 μm) and sediment layers below 1 cm depth had been included. We suspect that the number of foraminiferal species in UK-1 Stratum A alone probably exceeds 1000 with additional species being added along gradients of increasing water depth and decreasing productivity across the CCZ. At a global scale, the fact that the majority (80% of them monothalamids) of the 416 sediment-dwelling species we collected in UK-1 Stratum A were new to science challenges estimates of how many species of living benthic foraminifera, described and undescribed, exist in the global ocean. Published estimates range from 3,200^76^ to 10,000–12,000^77^, the most recent figure being 7,200^78^. However, these numbers overlook the existence of many undescribed monothalamids in the deep sea, as evidenced by the present and earlier studies^25^, as well as in coastal habitats^79^, in addition to the numerous unknown deep-sea monothalamid MOTUs revealed by environmental DNA surveys^54^,^69^. Although caution must be exercised when extrapolating from local to global diversity^80^, it seems likely that current estimates of global foraminiferal diversity are too low, possibly by a factor of at least two or three.

(5) This study is based on samples collected over a period of 12 days (October 10–21, 2013) and takes no account of faunal changes (e.g. in species dominance) occurring over seasonal, inter-annual and decadal time scales among various faunal groups, including foraminifera^81^. Longer-term faunal trends that span the policy and management timescales for deep-sea mining are particularly important to understand and will require repeated sampling over a period of years at particular sites within the CCZ.

## Methods

### Study area

The samples analysed in the present study were collected at around 4,080 m water depth in a 30 by 30 km stratum (UK-1 Stratum A) in the northern part of the UK-1 contract area (Table 1), located at the eastern end of the CCZ. Mean annual particulate organic carbon flux to the abyssal seafloor in this region is about 1 gC.m^−2^.y^−1^, double that in the western CCZ^82^. During the sampling campaign, multibeam bathymetric surveys revealed NNW to SSE ridges and valleys ranging between about 3,900 and 4,400 m water depth in our investigated area. Bottom water was fully oxic (dissolved oxygen ~3.2 ml.l^−1^), with a temperature of ~2 °C^50^. Abyssal current velocities were below sediment erosion thresholds throughout the sampling campaign (unpublished data, ABYSSLINE Project).

### Practical challenges posed by the study of abyssal foraminifera

More than a third (39%) of monothalamids in our samples were fragments, a pattern consistent with earlier studies of Pacific foraminifera based on fractions >297 μm^12^,^56^,^83^, >42 μm^56^ and >32 μm^13^,^27^,^56^. The fragments were dominated (69%) by agglutinated tubes and komokiaceans. We followed the approach of Nozawa *et al*.^13^ by counting fragments and complete individuals separately in order to avoid inflating foraminiferal densities. Where it was difficult to distinguish between fragments and complete individuals, we erred on the side of caution by regarding doubtful specimens as fragments. Nevertheless, since only 23 species were not represented by complete specimens (Table 3), fragments had a limited impact on diversity estimates.

We adopted strict criteria in order to do distinguish between ‘live’ and dead foraminiferal tests. Around half of all species in our samples accumulated stercomata (waste pellets), a common feature of deep-sea monothalamids^8^,^29^,^32^. The protoplasm to body volume ratio is often low in these taxa, the protoplasm being diffuse and often obscured by masses of stercomata^9^. The stercomata may persist in dead specimens, eventually decaying into grey ‘sediment’. All tests that appeared live were examined on glass slides in glycerol under a high-power compound microscope to ensure that they contained fresh stercomata, where present, as well as protoplasm.

The vast majority (90.1%) of the 416 species recognised during this study, including almost all (96.2%) of the 317 monothalamid species, were undescribed. Moreover, many of these undescribed species were rare, making comparisons with other studies difficult. The prevalence of rare undescribed species is a normal feature of the deep-sea benthos^44^,^84^ but in the case of monothalamids, it is sometimes compounded by intraspecific morphological plasticity^85^,^86^. We mitigated these challenges as far as possible by photographing every specimen extracted from the samples, and by careful comparison (on the same slide) of those from the same or different samples that appeared similar. We believe that this careful approach has minimised the uncertainties around species recognition.

### Sample collection and processing

Samples were collected during the ABYSSLINE 01 cruise (AB01, R/V *Melville*, cruise MV1313, 3–27 October 2013) at 5 sites across UK-1 Stratum A. At each site, one core was selected from those recovered by a megacorer (BCMEGA OSIL Bowers & Connelly type) equipped with twelve 10-cm diameter core tubes (78.6 cm^2^ surface area). As soon as possible after recovery, the cores were sliced into horizontal layers every 0.5 cm until 2 cm depth, and then every 1 cm from 2 to 10 cm depth. Each sediment layer was preserved in a plastic bottle with 10% formalin buffered with sodium borate. For the present investigation, only the 0–0.5 and 0.5–1 cm layers were analysed.

In the laboratory, the upper two sediment layers of 3 samples (MC02, MC04, MC07) were analysed in their entirety. Because this proved very time consuming, the 0–0.5 and 0.5–1.0 cm layers of the two remaining samples were each split into 1/8^th^ fractions using a wet splitter^87^ and all foraminifera from complete splits were sorted. The number of splits analysed (2/8^th^ for MC11, 3/8^th^ for MC09) was enough to yield a minimum of 300 individuals. Samples (either complete layers or splits) were sieved through 150 and 300 μm mesh screens. Each of these sieve fractions (i.e. 150–300 μm and >300 μm) was stained overnight with Rose Bengal (1 g in 1 litre of tap water) and all stained foraminifera, including the soft-shelled species as well as obviously stained forms living inside radiolarian tests^20^, were hand-picked from the residues in water under a binocular microscope. In order to ensure that stained specimens contained protoplasm and/or fresh stercomata, they were placed on a glass cavity slide in glycerol for observation under an Olympus BH-2 compound microscope.

Specimens were identified at the genus and species level when possible, or classified into informal morphospecies on the basis of test morphology and wall structure. Photographs of most specimens were taken using a SLR digital camera (Canon EOS 350D) fitted to an Olympus stereomicroscope. Where necessary, photographs were taken at different focal depths and combined into fully focused images using the open source image processing software package Combine ZP. Representative images of each species were assembled on PowerPoint slides in order to aid their subsequent recognition. Confirmation that specimens from different samples represented the same species often required them to be directly compared under the microscope. The cavity slides were left uncovered so that individual tests could be moved between slides for this purpose.

### Statistical analyses and diversity estimation

Statistical analyses and diversity estimations were computed using the open source software EstimateS (Version 9)^88^. Based on complete specimens only (either live only and live–dead combined), we calculated for each sample the Fisher alpha (α), Shannon (H, using natural logarithm) and Evenness (E) indices, and built individual-based rarefaction curves^89^. Since the curves had not reached an asymptote, we extrapolated these up to 1,500 individuals^89^, and calculated the Chao1 to estimate the expected total number of species for each sample. Because monothalamous benthic foraminifera tend to be overlooked in foraminiferal studies, we performed additional statistical analyses and diversity estimations on two kinds of partial assemblages, 1) ‘familiar’ species and 2) fossilising species. ‘Familiar’ species are those that we considered would remain intact in samples preserved in 95% ethanol and/or dried, and therefore included all multichambered species (fossilising and non-fossilising) and most robust monothalamids. Fossilising species were selected following Mackensen *et al*.^14^, and included all calcareous taxa (robertinids, rotaliids, lagenids, miliolids) and one agglutinated species using a calcareous cement (*Eggerella bradyi*).

Based on complete live and dead specimens combined, we built rarefaction curves for the 5 pooled samples (sample-based), based on the number of complete (live and dead combined) specimens. This method estimates the expected number of species in t pooled samples, given the reference sample^89^. Since the curve had not reached an asymptote with our 5 samples, we extrapolated the curve up to 15 samples^89^, and calculated diversity estimators in order to assess the expected total number of species of the 5 pooled samples. Since our 5 samples were not comparable in size (3 complete samples, 2 split samples), we followed the recommendations of Hortal *et al*.^90^ and calculated the Chao1, Abundance Coverage-based Estimator (ACE), Jacknife 1 and Jacknife 2. The abundance-based estimators Chao1 and ACE were calculated based on the number of complete specimens, live and dead combined, while Jacknife 1 and 2, which are incidence-based estimators, were calculated based on the occurrence of complete specimens and fragments, live and dead combined.

## Additional Information

**How to cite this article:** Goineau, A. and Gooday, A. J. Novel benthic foraminifera are abundant and diverse in an area of the abyssal equatorial Pacific licensed for polymetallic nodule exploration. *Sci. Rep.* **7**, 45288; doi: 10.1038/srep45288 (2017).

**Publisher's note:** Springer Nature remains neutral with regard to jurisdictional claims in published maps and institutional affiliations.

## Supplementary Material

Supplementary Information

## Figures and Tables

**Table 1:** Station data for the five investigated sampling sites in the UK-1 contract area.

Sampling sites	Samples	Latitude (°N)	Longitude (°W)	Water depth (m)
B	MC02	13°50.792′	116°37.59′	4079
D	MC04	13°57.796′	116°34.093′	4084
G	MC07	13°45.706′	116°27.601′	4111
H	MC09	13°53.299′	116°41.399′	4150
J	MC11	13°54.104′	116°35.401′	4166

MC = megacorer.

**Table 2:** Abundance of live complete, live fragmented, dead complete and dead fragmented foraminiferal specimens assigned to major groups (mixture of formal taxa and informal morphology-based groupings).

Abbreviation	Morphological groupings	Live complete			Live fragments			Dead complete			Dead fragments			Total complete		Total fragments	
		N	Indet.	%	N	Indet.	%	N	Indet.	%	N	Indet.	%	N	%	N	%
Multichambered																	
Rob	Robertinids	1	0	0.03	0	0	0.0	0	0	0.0	0	0	0.0	1	0.03	0	0.0
Rot	Rotaliids	71	3	2.7	0	0	0.0	40	0	4.6	0	0	0.0	114	3.2	0	0.0
Lag	Lagenids	5	0	0.2	0	0	0.0	4	0	0.5	0	0	0.0	9	0.2	0	0.0
Mil	Miliolids	20	1	0.8	0	0	0.0	3	0	0.3	0	0	0.0	24	0.7	0	0.0
Amm	Ammodiscids	3	0	0.1	0	0	0.0	32	0	3.7	0	0	0.0	35	1.0	0	0.0
Horm	Hormosinids	180	8	6.8	9	3	0.8	160	19	20.8	23	4	7.3	367	10.2	39	2.2
Troc	Trochamminids	11	0	0.4	0	0	0.0	48	4	6.0	0	0	0.3	63	1.7	0	0.0
MAF	Various MAF	29	2	1.1	0	0	0.0	102	0	11.8	1	0	0.0	133	3.7	1	0.1
	**Total multichambered**	**320**	**14**	**12**.**2**	**9**	**3**	**0**.**8**	**389**	**23**	**47**.**9**	**24**	**4**	**7**.**6**	**746**	**20**.**7**	**40**	**2**.**2**
	**Density per 10 cm^2^**	**11**.**0**	**0**.**8**		**0**.**2**	**0**.**1**		**13**.**1**	**0**.**8**		**1**.**6**	**0**.**2**		**25**.**8**	**0**.**7**	**2**	**0**.**1**
Monothalamids																	
Nod-l	*Nodellum*-like	74	0	2.7	2	0	0.1	21	1	2.6	9	0	2.4	96	2.7	11	0.6
L&F	*Lagenammina* & Flasks	205	4	7.6	0	0	0.0	128	4	15.3	0	0	0.0	341	9.5	0	0.0
Sac	Saccamminids	59	1	2.2	0	0	0.0	10	0	1.2	0	0	0.0	70	1.9	0	0.0
Sph	Spheres	311	337	23.6	0	0	0.0	23	1	2.8	0	0	0.0	672	18.6	0	0.0
Org	Organic-walled	56	5	2.2	2	0	0.1	0	0	0.0	0	0	0.0	61	1.7	2	0.1
Hyp	Hyperamminids	6	0	0.2	0	0	0.0	10	0	1.2	0	0	0.0	16	0.4	0	0.0
Tub	Tubes	599	2	21.9	493	31	36.7	166	0	19.3	169	10	48.5	767	21.3	703	39.1
Ch-l	Chain-like	50	0	1.8	187	0	13.1	11	0	1.3	24	0	6.5	61	1.7	211	11.7
Unc	Unclassified	327	3	12.0	354	4	25.1	42	0	4.9	46	0	12.5	372	10.3	404	22.5
Kom-l	Komokiacean-like	156	0	5.7	40	0	2.8	22	0	2.6	9	0	2.4	178	4.9	49	2.7
Komokiaceans																	
Bac	Baculellidae	164	0	6.0	213	0	14.9	3	0	0.3	21	0	5.7	167	4.6	234	13.0
Kom	Komokiidae	41	3	1.6	62	29	6.4	7	0	0.8	21	32	14.4	51	1.4	144	8.0
	**Total monothalamids**	**2048**	**355**	**87**.**5**	**1353**	**64**	**99**.**2**	**443**	**6**	**52**.**1**	**299**	**42**	**92**.**4**	**2852**	**79**.**1**	**1758**	**97**.**8**
	**Density per 10 cm^2^**	**85**.**7**	**14**.**2**		**50**.**7**	**4**.**6**		**15**.**5**	**0**.**3**		**13**.**8**	**1**.**6**		**115**.**7**	**3**.**2**	**71**	**3**.**9**
Gr	Gromiids	9	0	0.3	0	0	0.0	0	0	0.0	0	0	0.0	9	0.2	0	0.0
	**Total**	**2377**	**369**		**1362**	**67**		**832**	**29**		**323**	**46**		**3607**		**1798**	
	**Total density per 10 cm^2^**	**96**.**9**	**15**.**1**		**51**.**0**	**4**.**7**		**28**.**6**	**1**.**1**		**15**.**4**	**1**.**8**		**141**.**7**		**72**.**8**	

‘N’ and ‘Indet.’ = the number of individuals assigned to species and the number of indeterminate specimens, respectively. ‘%’ = their relative abundance (assigned to species and indeterminate combined). Data are based on the total number of specimens in the 0–1 cm layer (>150-μm fraction) of the 5 samples combined. MAF = multichambered agglutinated foraminifera.

**Table 3:** Species richness of live complete, live fragmented, dead complete, dead fragmented and total assemblages into the top 0–1 cm layer of the 5 samples combined.

Morphological groupings	Live complete			Live fragments			Dead complete			Dead fragments			Total			Number described	
	S_Tot._	%	S_add._	S_Tot._	%	S_add._	S_Tot._	%	S_add._	S_Tot._	%	S_add._	S_Tot._	%	Singl.	Species	Genus
Multichambered																	
Robertinids	1	0.3	1	—	—	—	—	—	—	—	—	—	1	0.2	1	1	—
Rotaliids	16	4.5	11	—	—	—	11	8.4	3	—	—	—	19	4.6	8	9	7
Lagenids	1	0.3	1	—	—	—	4	3.1	4	—	—	—	5	1.2	4	—	1
Miliolids	5	1.4	4	—	—	—	1	0.8	—	—	—	—	5	1.2	1	1	3
Ammodiscids	2	0.6	—	—	—	—	3	2.3	1	—	—	—	3	0.7	0	2	1
Hormosinids	28	7.9	9	1	1.2	—	19	14.5	1	4	9.3	—	29	7.0	6	6	23
Trochamminids	8	2.3	2	—	—	—	10	7.6	4	—	—	—	12	2.9	3	2	7
Various MAF	9	2.5	1	—	—	—	21	16.0	13	1	2.3	—	22	5.3	8	10	12
**Total multichambered**	**70**	**19**.**8**	**29**	**1**	**1**.**2**	**0**	**69**	**52**.**7**	**26**	**5**	**11**.**6**	**0**	**96**	**23**.**1**	**31**	**31**	**54**
Monothalamids																	
*Nodellum*-like	12	3.4	8	1	1.2	—	4	3.1	—	2	4.7	—	12	2.9	6	1	7
*Lagenammina* & Flasks	13	3.7	2	—	—	—	11	8.4	—	—	—	—	13	3.1	0	2	7
Saccamminids	18	5.1	14	—	—	—	5	3.8	1	—	—	—	19	4.6	9	—	2
Spheres	64	18.0	60	—	—	—	6	4.6	2	—	—	—	66	15.9	30	2	4
Organic-walled	17	4.8	16	1	1.2	—	—	—	—	—	—	—	17	4.1	8	1	5
Hyperamminids	3	0.8	2	—	—	—	2	1.5	1	—	—	—	4	1.0	0	1	3
Tubes	52	14.6	23	39	46.4	5	14	10.7	—	18	41.9	2	65	15.6	14	1	10
Chain-like	10	2.8	3	6	7.1	2	5	3.8	2	3	7.0	—	14	3.4	3	—	—
Unclassified	38	10.7	28	10	11.9	1	7	5.3	2	3	7.0	—	42	10.1	11	—	2
Komokiacean-like	21	5.9	14	6	7.1	—	3	2.3	—	4	9.3	—	22	5.3	2	—	—
Komokiaceans																	
Baculellidae	19	5.4	9	13	15.5	2	2	1.5	1	3	7.0	—	23	5.5	2	2	12
Komokiidae	15	4.2	11	7	8.3	2	3	2.3	1	5	11.6	—	20	4.8	5	—	7
**Total monothalamids**	**282**	**79**.**4**	**190**	**83**	**98**.**8**	**12**	**62**	**47**.**3**	**10**	**38**	**88**.**4**	**2**	**317**	**76**.**2**	**90**	**10**	**59**
Gromiids	3	0.8	3		0.0	—		0.0	—		0.0	—	3	0.7	1	—	—
**Total**	**355**		**222**	**84**		**12**	**131**		**36**	**43**		**2**	**416**		**122**	**41**	**113**

For each morphological grouping, ‘S_Tot._’ = the total number of species. ‘%’ = the relative contribution to the total diversity within each assemblage, and ‘S_add._’ = the number of species occurring only in the corresponding assemblage, ‘Singl.’ = the number of singletons. The two right-hand columns indicate the number of taxa identified down to a described species or a genus (but undescribed species). MAF = multichambered agglutinated foraminifera.

**Table 4:** Diversity indices based on live complete specimens, and on live and dead complete (combined), computed for each sample based on the entire assemblage (‘Entire sp.’, i.e. all the species), ‘familiar’ species (‘Fam. sp.’) and fossilising species (‘Fossil. sp.’).

Component	Sample	Assemblage	N	D	S	%S_Tot_	E(S100)	Chao1	%	Singl.	Doubl.	H	α	E
Live complete														
	MC02	Entire sp.	506	64.4	115	32.4	48	176 ± 23	65.5	51	20	3.93	46.4	0.44
		Fam. sp.	159	20.2	53	40.5	42	82 ± 15	64.4	26	10	3.41	27.8	0.57
		Fossil. sp.	26	3.3	7	31.8	8	7 ± 1	95.6	2	2	1.53	3.1	0.66
	MC04	Entire sp.	355	45.2	108	30.4	54	161 ± 20	67.0	50	22	4.13	52.9	0.58
		Fam. sp.	121	15.4	46	35.1	42	65 ± 11	70.7	22	11	3.45	27.1	0.69
		Fossil. sp.	20	2.5	11	50.0	34	25 ± 15	39.1	9	1	1.94	10.0	0.63
	MC07	Entire sp.	848	108.0	182	51.3	57	273 ± 28	66.6	77	31	4.42	71.1	0.46
		Fam. sp.	383	48.8	83	63.4	43	134 ± 23	61.9	37	12	3.69	32.6	0.48
		Fossil. sp.	43	5.5	14	63.6	19	19 ± 5	73.2	7	3	2.13	7.2	0.60
	MC09	Entire sp.	431	146.3	143	40.3	60	233 ± 29	61.3	73	28	4.42	74.8	0.58
		Fam. sp.	166	56.4	51	38.9	38	101 ± 26	50.3	29	7	3.25	25.1	0.51
		Fossil. sp.	5	1.7	4	18.2	8	5 ± 2	76.9	3	1	1.33	9.3	0.95
	MC11	Entire sp.	237	120.7	86	24.2	54	134 ± 20	64.2	41	16	4.08	48.5	0.69
		Fam. sp.	55	28.0	27	20.6	38	49 ± 15	54.8	17	5	2.97	21.0	0.72
		Fossil. sp.	2	1.0	1	4.5	1	1 ± 0	100.0	0	1	0	0.8	1.00
Live and dead complete														
	MC02	Entire sp.	741	94.3	142	36.1	50	229 ± 31	62.0	61	20	4.13	52.2	0.44
		Fam. sp.	293	37.3	74	45.4	43	122 ± 23	60.7	33	10	3.68	31.9	0.54
		Fossil. sp.	33	4.2	9	30.0	14	12 ± 4	75.6	4	1	1.7	4.08	0.61
	MC04	Entire sp.	557	70.9	135	34.4	53	194 ± 20	69.6	59	28	4.20	56.7	0.50
		Fam. sp.	287	36.5	69	42.3	40	98 ± 14	70.4	31	15	3.50	28.8	0.48
		Fossil. sp.	29	3.7	16	53.3	31	29 ± 11	54.6	11	3	2.43	14.7	0.71
	MC07	Entire sp.	1101	140.2	208	52.9	58	293 ± 25	71.0	82	38	4.55	75.9	0.45
		Fam. sp.	595	75.8	107	65.6	46	155 ± 20	69.1	42	17	3.96	38.1	0.49
		Fossil. sp.	66	8.4	17	56.7	21	26 ± 8	65.7	9	3	2.1	7.4	0.48
	MC09	Entire sp.	531	180.3	166	42.2	63	249 ± 25	66.5	77	34	4.60	82.9	0.60
		Fam. sp.	259	87.9	74	45.4	45	120 ± 21	61.9	35	12	3.73	34.6	0.57
		Fossil. sp.	14	4.7	8	26.7	14	11 ± 4	72.1	5	2	1.87	7.8	0.81
	MC11	Entire sp.	279	142.1	98	24.9	57	147 ± 20	66.5	45	19	4.21	53.8	0.68
		Fam. sp.	81	41.3	37	22.7	41	58 ± 12	64.0	20	8	3.32	26.3	0.75
		Fossil. sp.	2	1.0	1	3.3	1	1 ± 0	100.0	0	1	0	0.8	1.00

N = no. individuals; D = : no. individuals per 10 cm^2^; S = no. species; %S_Tot_ = proportion of species recognised in individual samples relatively to the total number of species (in the corresponding assemblage) in the 5 combined samples; E(S100) = estimated number of species for 100 individuals; Chao1 = estimated total number of species; % = proportion of diversity recognised in individual samples relatively to Chao1; Singl. = no. singletons; Doubl. = no. doubletons; H = Shannon index; α = Fisher alpha; E = Evenness index.
