# Supplementary material for: Novel benthic foraminifera are abundant and diverse in an area of the abyssal equatorial Pacific licensed for polymetallic nodule exploration
Source: Sci Rep. 2017 Apr 6;7:45288. doi: 10.1038/srep45288 (PMC5382569; doi:10.1038/srep45288)

Novel benthic foraminifera are abundant and diverse in an area of the abyssal equatorial Pacific licensed for polymetallic nodule exploration.

Aurélie Goineau*, Andrew J. Gooday

National Oceanography Centre, University of Southampton, Waterfront Campus, European Way, Southampton SO14 3ZH, United Kingdom

* Corresponding author: A.Goineau@noc.ac.uk – +44 (0) 23 8059 6333

Supplementary information

Supplementary Table S1 (p. II): Species numbers based on other published Pacific studies.

Supplementary Table S2 (p. III): Number of live complete (‘L’), live fragmented (‘LFrag’), dead complete (‘D’) and dead fragmented (‘DFrag’) specimens of each morphological grouping in each sample, normalised to a surface area of 10 cm².

Supplementary Table S3 (p. IV): Taxonomic composition (% of morphological groupings) of live complete (‘L’), live fragmented (‘LFrag’), dead complete (‘D’) and dead fragmented (‘DFrag’) specimens that were placed into species ('identified') or higher taxa level ('indeterminate') in each sample.

Supplementary Table S4 (p. V): 30 top-ranked species represented by complete specimens and by fragmented specimens (live and dead combined). Group = formal or informal taxonomical/morphological grouping of the species.

Supplementary Material S1 (pp. VI–XV): Description of the top-ranked species among complete specimens and fragments, live and dead combined.

Supplementary Material S2 (pp. XVI–XVIII): Taxonomic list of taxa assigned to a named species that do not appear in the top-ranked species among complete specimens and fragments (live and dead combined). References to plates and figures in literature are provided.

Supplementary Material S3 (pp. XIX–XX): List of references cited in Supplementary Table S1 and Supplementary Materials S1 and S2.

Supplementary Figure S1 (p. XXI): Species ranked by abundance based on complete (live and dead) specimens (all samples combined). For information, the level of 5 specimens is indicated by a dotted line.

Supplementary Figures S2 to S11 (pp. XXII–XXXI): Reflected and transmitted light photographs of the most abundant species represented by complete specimens or fragments (live and dead combined).

Supplementary Table S1: Species numbers based on published Pacific data. Abbreviations: ND: no data (i.e. this faunal component was not included in the study or was not counted separately); Komok.: Komokiaceans; Nodel.: *Nodellum* ; Ammod.: Ammodiscids; Spiril.: Spirillinids; Miliol.: Miliolids; Horm.: Hormosinids; MAF: other Multichambered Agglutinated Foraminifera; Rotal.: Rotaliids; Robert.: Robertinids; Lagen.: Lagenids; BC: Box core; MuC: Multicorer.

									**Monothalamids**				**Tubulothalamea**		**Globothalamea**					
**Region of Pacific**	**Area/Site**	**Coordinates**	**Depth (m)**	**No. sites/samples**	**Sediment layer**	**Sieve**	**Stained vs Dead**	**No. specimens**	**Xenos**	**Komok.**	**Nodel.**	**Others**	**Ammod. +Spiril.**	**Miliol.**	**Horm.**	**MAF**	**Rotal. +Robert.**	**Lagen.**	**TOTAL**	**Reference**
**Monographs**																				
N. Pacific	Entire area		Coastal to abyssal	Thousands			Live + Dead	Not known	3	0	1	52	14	115	14	115	208	175	702*	Cushman (1910–1917)
Pacific	Entire ocean		1–10,002	>1,620			Live + Dead?	Not known	3	6**	1	178	18+26	322	18	322	666	227	1796	Saidova (1975)
**Studies based entirely or mainly on hard-shelled taxa**																				
N. Pacific	Aleutian Terrace	54°33'–57°17'N, 154°27'–157°59'W	10–121	3	0–2 cm	75 µm	Stained + Dead	690D + 27L***	ND	ND	0	0	0	1	0	5	28	1	35	Smith (1973)
N. Pacific	Aleutian Trench	50°24'N, 176°30'E	7,000–7,230	2	0–2 cm	75 µm	Stained + Dead	1,652D + 4L	ND	ND	2	6	1	1	6	24	4	0	44	Smith (1973)
N. Pacific	Deep-sea Plain	23°22'–59°50'N, 155°24'–177°54'W	2,410–6,560	19	0–2 cm	75 µm	Stained + Dead	3805D + 150L	ND	ND	0	6	1	4	6	25	13	0	55	Smith (1973)
W. Eq. Pacific	Ontong Java Plateau	155–163°W	1,611–4,332	29	0–5 cm	125 µm	Unstained	~5234	ND	ND	0	6	1	6	3	19	120	9	164	Burke (1981)
NE Pacific	Hess Rise	34°'N, 179°E	2,167–3,354	4	0–2 cm	63 µm	Stained	527	ND	ND	0	4	0	9	4	13	21	8	59	Ohkushi and Natori (2001)
NE Pacific	Suiko Seamount	45°'N, 170°E	1,811–1,995	3	0–2 cm	63 µm	Stained	407	ND	ND	1	4	1	9	4	7	21	5	52	Ohkushi and Natori (2001)
E. Eq. Pacific	Guatemala Basin	06°27'–10°38'N, 92°00'–100°31'W	3,350–4,040	9	0–1 cm (0–2 cm)	42 µm	Stained + Dead	100–350 per sample	ND	ND	0	15	2	ND	10	29	ND	ND	65	Burmistrova et al. (2007)
E. Eq. Pacific	Area I	10°N, 140°W	4,918–4,970	6	0–1 cm (0–2 cm)	42 µm	Stained + Dead	100–350 per sample	ND	ND	0	10	2	ND	8	13	ND	ND	33	Burmistrova et al. (2007)
E. Eq. Pacific	Area II	13°30'N, 132°58'W	4,665–4,935	3	0–1 cm (0–2 cm)	42 µm	Stained + Dead	100–350 per sample	ND	ND	0	17	1	ND	6	17	ND	ND	41	Burmistrova et al. (2007)
NE Pacific	Station M	34°50' N, 123°00' W	3953	8 push cores	0–5 cm, 0–3 cm	63 µm	Stained + Dead	3638	ND	ND	0	14	3	7+	10+	11	22+	2+	84+	Enge et al. (2012)
NE Pacific	Station M	34°50' N, 123°00' W	3953	6 push cores	0–3 cm	63 µm	Stained	2470	ND	ND	0	4	68 agglut. (excluding 4 soft-walled saccamminids) + 45 calcareous						117	Enge et al. (2011)
**Studies based largely or entirely on soft-shelled monothalamids**																				
N. Pacific	Climax II site	58°N, 155°E	5,100 m	5 BCs	0–2 cm, 0–10 cm	297 µm	Stained + Dead	36,017 (incl. fragments)	ND	ND	ND	ND	ND	ND	ND	ND	ND	ND	179	Bernstein et al. (1978)
N. Pacific	Climax II site	58°N, 155°E	5,100 m	5 BC subcores	0–10 cm	297 µm	Stained + Dead	7984	ND	56	85 sp. excluding Komokiacea								141	Bernstein et al. (1978)
N. Pacific	Climax II site	58°N, 155°E	5,101 m	1 BC subcore	0–2 cm	297 µm?	Stained + Dead	1302	ND	ND	ND	ND	ND	ND	ND	ND	ND	ND	40	Bernstein and Meador (1979)
E. Eq. Pacific	Kaplan East (CCZ)	15°N, 119°W	4,089–4,159	20 MuC subcores	0–1 cm	32 µm	Stained	983 (+4,438 indet) complete	0	ND	ND	ND	ND	ND	ND	ND	ND	ND	168	Nozawa et al. (2006)
E. Eq. Pacific	Kaplan East (CCZ)	15°N, 119°W	4,089–4,159	20 MuC subcores	0–1 cm	32 µm	Stained	8,075 fragments	0	47	ND	37	ND	ND	ND	ND	ND	ND	84	Nozawa et al. (2006)
E. Eq. Pacific	IOM claim (CCZ)	11°N, 119° W	4,380–4,410	3 MuCs	0–6 cm	250 µm	Stained	ND	4	46	ND	52	ND	ND	ND	ND	ND	ND	102	Kamenskaya et al. (2012)

***D: dead, L: live　　**See Gooday et al. (2007, Table 1)　　*Includes 'varieties'

Supplementary Table S2: Number of live complete ('L'), live fragmented ('L_{Frag}'), dead complete ('D') and dead fragmented ('D_{Frag}') specimens of each morphological grouping in individual samples, normalised to a surface area of 10 cm². Values combine specimens that were placed into species ('identified') or higher taxa level ('indeterminate').

Samples	MC02				MC04				MC07				MC09				MC11			
Morphological groupings	L	L_{Frag}	D	D_{Frag}	L	L_{Frag}	D	D_{Frag}	L	L_{Frag}	D	D_{Frag}	L	L_{Frag}	D	D_{Frag}	L	L_{Frag}	D	D_{Frag}
<u>Multichambered</u>																				
Robertinids	0	0	0	0	0	0	0	0	0.1	0	0	0	0	0	0	0	0	0	0	0
Rotaliids	2.7	0	0.8	0	2.0	0	0.9	0	3.4	0	2.4	0	2.7	0	2.7	0	1.0	0	0	0
Lagenids	0	0	0.1	0	0.1	0	0.3	0	0.5	0	0.1	0	0	0	0	0	0	0	0	0
Miliolids	0.6	0	0	0	0.4	0	0	0	1.5	0	0.3	0	0.3	0	0.3	0	0	0	0	0
Ammodiscids	0.1	0	0.1	0	0.0	0	0.6	0	0.1	0	2.9	0	0.3	0	0.7	0	0	0	0.5	0
Hormosinids	2.9	0	4.8	0.3	3.7	0	4.5	0.1	11.5	1.5	9.8	0.5	12.2	0	7.5	5.4	5.1	0	3.6	2.0
Trochamminids	0.4	0	1.8	0	0.1	0	2.0	0	0.5	0	2.2	0	0.7	0	1.4	0	0.5	0	0.5	0
Various MAF	1.5	0	2.7	0	0.8	0	1.7	0	0.9	0	5.6	0	1.0	0	6.8	0	1.5	0	2.0	0.5
**Total identified**	**8.3**	**0**	**9.3**	**0.3**	**7.1**	**0**	**9.3**	**0.1**	**18.1**	**1.1**	**22.5**	**0.3**	**14.6**	**0**	**18.0**	**4.7**	**7.1**	**0**	**6.6**	**2.5**
**Total indeterminate**	**0**	**0**	**1.0**	**0.0**	**0**	**0**	**0.6**	**0**	**0.5**	**0.4**	**0.8**	**0.3**	**2.7**	**0**	**1.4**	**0.7**	**1.0**	**0**	**0**	**0**
<u>Monothalamids</u>																				
Nodellum -like	1.7	0	0	0	1.7	0.3	1.7	0.5	3.2	0	0.8	0.6	6.1	0	1.0	0	2.5	0	0	0
Lagenammina & Flasks	5.2	0	2.0	0	2.4	0	8.5	0	12.3	0	3.1	0	12.9	0	6.4	0	7.1	0	3.1	0
Saccamminids	2.7	0	0	0	1.3	0	0	0	2.0	0	0.4	0	4.1	0	0.7	0	0.5	0	2.5	0
Spheres	17.4	0	0	0	10.8	0	1.7	0	31.3	0	0.5	0	37.7	0	1.7	0	35.1	0	1.0	0
Organic-walled	1.9	0.3	0	0	0.9	0	0.0	0	2.9	0	0	0	4.1	0	0	0	2.0	0	0	0
Hyperamminids	0	0.0	0.4	0	0	0	0.1	0	0.8	0	0.1	0	0	0	1.7	0	0	0	0	0
Tubes	23.7	8.1	14.1	1.1	8.4	8.0	3.4	5.7	22.0	25.2	1.8	5.0	40.7	30.2	3.1	9.5	28.5	56.0	2.5	29.5
Chain-like	1.5	0.5	0.9	0	2.3	0.9	0.3	0.6	0.4	21.2	0.1	2.0	4.7	2.4	0.3	1.0	1.5	1.0	0	0
Unclassified	5.7	6.0	3.1	0	8.0	2.7	0.3	0.8	17.3	34.6	1.1	4.8	15.3	0.7	1.4	0	20.4	8.1	1.5	1.0
Komokiacean-like	1.5	0	0	0.4	4.7	3.6	0	0.3	6.2	1.0	1.8	0.4	12.2	0	0.3	0	11.2	2.0	3.6	0.5
Komokiaceans																				
Baculellidae	7.0	6.6	0.1	1.7	2.5	7.3	0	0.5	3.6	9.3	0	0	8.8	3.7	0.3	0.3	17.8	10.2	0.5	1.5
Komokiidae	0	0.3	0	0.3	0.9	0.4	0.8	1.3	1.7	4.2	0	4.2	5.1	10.5	0.3	1.7	4.6	11.2	0	1.5
**Total identified**	**55.9**	**21.6**	**20.6**	**3.4**	**38.0**	**22.9**	**16.4**	**9.7**	**88.9**	**93.3**	**9.7**	**13.2**	**131.6**	**43.4**	**15.9**	**8.5**	**113.5**	**72.3**	**14.8**	**34.1**
**Total indeterminate**	**12.5**	**0.1**	**0**	**0**	**6.0**	**0.1**	**0.3**	**0**	**14.8**	**2.3**	**0**	**3.8**	**20.0**	**4.1**	**1.4**	**4.1**	**17.8**	**16.3**	**0.0**	**0.0**
<u>Gromiids</u>	0.3	0	0	0	0	0	0	0	0.9	0	0	0	0	0	0	0	0	0	0	0
**Total identified**	**64.4**	**21.6**	**29.9**	**3.7**	**45.2**	**22.9**	**25.7**	**9.8**	**107.9**	**94.4**	**32.2**	**13.5**	**146.2**	**43.4**	**33.9**	**13.2**	**120.6**	**72.3**	**21.4**	**36.6**
**Total indeterminate**	**12.5**	**0.1**	**1.0**	**0.0**	**6.0**	**0.1**	**0.9**	**0.0**	**15.3**	**2.7**	**0.8**	**4.1**	**22.7**	**4.1**	**2.7**	**4.7**	**18.8**	**16.3**	**0.0**	**0.0**
**Total**	**76.8**	**21.8**	**30.9**	**3.7**	**51.1**	**23.0**	**26.6**	**9.8**	**123.2**	**97.1**	**33.0**	**17.6**	**169.0**	**47.5**	**36.6**	**18.0**	**139.4**	**88.5**	**21.4**	**36.6**

Supplementary Table S3: Taxonomic composition (% of morphological groupings) of live complete (‘L’), live fragmented (‘L_{Frag}’), dead complete (‘D’) and dead fragmented (‘D_{Frag}’) specimens that were placed into species ('identified') or higher taxa level ('indeterminate') in each sample.

Samples	MC02				MC04				MC07				MC09				MC11			
Morphological groupings	L	L_{Frag}	D	D_{Frag}	L	L_{Frag}	D	D_{Frag}	L	L_{Frag}	D	D_{Frag}	L	L_{Frag}	D	D_{Frag}	L	L_{Frag}	D	D_{Frag}
Multichambered																				
Robertinids	0	0	0	0	0	0	0	0	0.1	0	0	0	0	0	0	0	0	0	0	0
Rotaliids	3.5	0	2.5	0	4.0	0	3.3	0	2.8	0	7.3	0	1.6	0	7.4	0	0.7	0	0	0
Lagenids	0	0	0.4	0	0.2	0	1.0	0	0.4	0	0.4	0	0.0	0	0	0	0	0	0	0
Miliolids	0.8	0	0	0	0.7	0	0.0	0	1.2	0	0.8	0	0.2	0	0.9	0	0	0	0	0
Ammodiscids	0.2	0	0.4	0	0	0	2.4	0	0.1	0	8.9	0	0.2	0	1.9	0	0	0	2.4	0
Hormosinids	3.8	0	15.6	6.9	7.2	0	16.7	1.3	9.3	1.6	29.7	2.9	7.2	0	20.4	30.2	3.6	0	16.7	5.6
Trochamminids	0.5	0	5.8	0	0.2	0	7.7	0	0.4	0	6.6	0	0.4	0	3.7	0	0.4	0	2.4	0
Various MAF	2.0	0	8.6	0	1.5	0	6.2	0	0.7	0	17.0	0	0.6	0	18.5	0	1.1	0	9.5	1.4
**Total identified**	**10.8**	**0**	**30.0**	**6.9**	**13.9**	**0**	**34.9**	**1.3**	**14.7**	**1.2**	**68.3**	**1.4**	**8.6**	**0**	**49.1**	**26.4**	**5.1**	**0**	**31.0**	**6.9**
**Total indeterminate**	**0**	**0**	**3.3**	**0**	**0**	**0**	**2.4**	**0**	**0.4**	**0.4**	**2.3**	**1.4**	**1.6**	**0**	**3.7**	**3.8**	**0.7**	**0**	**0**	**0**
Monothalamids																				
Nodellum -like	2.2	0	0	0	3.2	1.1	6.2	5.2	2.6	0	2.3	3.6	3.6	0	2.8	0	1.8	0	0	0
Lagenammina & Flasks	6.8	0	6.6	0	4.7	0	32.1	0	10.0	0	9.3	0	7.6	0	17.6	0	5.1	0	14.3	0
Saccamminids	3.5	0	0	0	2.5	0	0	0	1.7	0	1.2	0	2.4	0	1.9	0	0.4	0	11.9	0
Spheres	22.7	0	0	0	21.1	0	6.2	0	25.4	0	1.5	0	22.3	0	4.6	0	25.2	0	4.8	0
Organic-walled	2.5	1.2	0	0	1.7	0	0	0	2.4	0	0	0	2.4	0	0	0	1.5	0	0	0
Hyperamminids	0	0	1.2	0	0	0	0.5	0	0.6	0	0.4	0	0	0	4.6	0	0	0	0	0
Tubes	30.8	37.4	45.7	31.0	16.5	34.8	12.9	58.4	17.9	26.0	5.4	28.3	24.1	63.6	8.3	52.8	20.4	63.2	11.9	80.6
Chain-like	2.0	2.3	2.9	0	4.6	3.9	1.0	6.5	0.3	21.9	0.4	11.6	2.8	5.0	0.9	5.7	1.1	1.1	0	0
Unclassified	7.5	27.5	9.9	0	15.7	11.6	1.0	7.8	14.1	35.6	3.5	27.5	9.0	1.4	3.7	0	14.6	9.2	7.1	2.8
Komokiacean-like	2.0	0	0	10.3	9.2	15.5	0	2.6	5.1	1.0	5.4	2.2	7.2	0	0.9	0	8.0	2.3	16.7	1.4
Komokiaceans																				
Baculellidae	9.1	30.4	0.4	44.8	5.0	31.5	0	5.2	2.9	9.6	0	0	5.2	7.9	0.9	1.9	12.8	11.5	2.4	4.2
Komokiidae	0	1.2	0	6.9	1.7	1.7	2.9	13.0	1.3	4.3	0	23.9	3.0	22.1	0.9	9.4	3.3	12.6	0	4.2
**Total identified**	**72.7**	**99.4**	**66.7**	**93.1**	**74.4**	**99.4**	**61.7**	**98.7**	**72.3**	**96.1**	**29.3**	**75.4**	**77.9**	**91.4**	**43.5**	**47.2**	**81.4**	**81.6**	**69.0**	**93.1**
**Total indeterminate**	**16.2**	**0.6**	**0**	**0**	**11.7**	**0.6**	**1.0**	**0.0**	**12.0**	**2.4**	**0**	**21.7**	**11.8**	**8.6**	**3.7**	**22.6**	**12.8**	**18.4**	**0**	**0**
Gromiids	0.3	0	0	0	0	0	0	0	0.7	0	0	0	0	0	0	0	0	0	0	0
**Total No. individuals identified**	**506**	**170**	**235**	**29**	**355**	**180**	**202**	**77**	**848**	**742**	**253**	**106**	**431**	**128**	**100**	**39**	**237**	**142**	**42**	**72**
**Total No. individuals indeterminate**	**98**	**1**	**8**	**0**	**47**	**1**	**7**	**0**	**120**	**21**	**6**	**32**	**67**	**12**	**8**	**14**	**37**	**32**	**0**	**0**
**Total No. individuals**	**604**	**171**	**243**	**29**	**402**	**181**	**209**	**77**	**968**	**763**	**259**	**138**	**498**	**140**	**108**	**53**	**274**	**174**	**42**	**72**
**Total No. species**	**115**	**12**	**48**	**7**	**108**	**25**	**52**	**16**	**182**	**48**	**71**	**17**	**143**	**20**	**46**	**11**	**86**	**32**	**22**	**13**

Supplementary Table S4: 30 top-ranked species represented by complete specimens and by fragmented specimens (live and dead combined). Group: formal or informal taxonomical/morphological grouping of the species (see Table 2 for abbreviations); N: total number of complete specimens or fragments; %: contribution of each species relative to the total number of complete specimens or fragments assigned to species in the 5 samples; Occur.: number of samples in which the species occurs; Top 10: number of samples where the species was ranked in the top 10 species. '*' indicates the species appearing in both top 30-ranked species.

Group	Rank	Species from complete specimens	N	%	Occur.	Top 10
Tub	1	Tube sp. 48	178	4.93	4	2
L&F	2	Lagenamminid sp. 4	147	4.08	5	4
Tub	3	Tube sp. 54*	127	3.52	5	4
Sph	4	Psammosphaerid sp. 19	95	2.63	5	3
Kom-l	5	Komokiacean-like sp. 20*	87	2.41	5	3
Horm	6	*Reophax scorpiurus*	68	1.89	5	2
Unc	7	Monothalamid sp. 17	61	1.69	5	2
Tub	8	Tube sp. 40*	59	1.64	5	2
Unc	9	Monothalamid sp. 19	58	1.61	5	2
L&F	10	Lagenamminid sp. 3	56	1.55	4	1
Rot	10	*Nuttalides umbonifera*	56	1.55	4	1
Tub	12	Tube sp. 9	52	1.44	1	1
L&F	13	*Lagenammina difflugiformis*	51	1.41	4	1
Nod-l	14	*Nodellum* sp .1	46	1.28	5	0
Horm	15	*Hormosinella guttifera*	41	1.14	5	0
MAF	15	?*Verneuilinulla propinqua*	41	1.14	4	0
Horm	17	*Reophax* sp. 2	39	1.08	5	1
Unc	18	Monothalamid sp. 27*	37	1.03	4	1
MAF	19	*Cribrostomoides subglobosa*	34	0.94	5	0
Bac	20	*Baculella globofera*	31	0.86	3	1
Horm	20	*Reophax* aff. *helenae*	31	0.86	4	1
Tub	20	Tube sp. 25	31	0.86	5	1
Unc	20	Monothalamid sp. 23	31	0.86	4	0
Sph	24	Psammosphaerid sp. 20	30	0.83	2	1
Tub	24	Tube sp. 44*	30	0.83	1	1
Tub	26	Chain sp. 13*	28	0.78	4	0
Bac	27	*Edgertonia floccula*	27	0.75	5	1
Horm	27	*Reophax* sp. 1	27	0.75	5	0
Amm	27	*Usbekistania charoides*	27	0.75	5	0
Tub	27	Tube sp. 32*	27	0.75	3	1
		**Total Top 30**	**1653**	**45.8**		
		**Total 5 stations**	**3607**			

Group	Rank	Species from fragments	N	%	Occur.	Top 10
Unc	1	Monothalamid sp. 32	195	10.85	3	3
Ch-l	2	Chain sp. 13*	160	8.90	3	2
Unc	3	Monothalamid sp. 27*	138	7.68	3	3
Bac	4	*Edgertonia* sp. 7	107	5.95	5	3
Tub	5	Tube sp. 43	98	5.45	5	4
Tub	6	Tube sp. 10	89	4.95	4	2
Tub	7	*Rhizammina* sp. 1	65	3.62	1	1
Bac	8	*Edgertonia* sp.4	61	3.39	5	4
Tub	9	Tube sp.56	59	3.28	3	1
Unc	10	Monothalamid sp. 33	40	2.22	1	1
Kom	11	*Reticulum* sp. 1	37	2.06	5	2
Tub	12	Tube sp. 44*	36	2.00	3	2
Tub	13	Tube sp. 32*	28	1.56	3	2
Tub	14	Tube sp. 57	27	1.50	2	1
Tub	15	Tube sp. 45	23	1.28	2	1
Tub	15	Tube sp. 46	23	1.28	1	1
Tub	17	Tube sp. 41	21	1.17	2	1
Bac	18	*Edgertonia* sp.5	19	1.06	4	1
Tub	19	Tube sp. 40*	18	1.00	4	0
Tub	19	Tube sp. 54*	18	1.00	3	2
Tub	21	*Bathysiphon* sp.2	15	0.83	2	1
Kom-l	22	Komokiacean-like sp. 20*	14	0.78	2	1
Kom	22	*Lana* sp. 1	14	0.78	2	1
Kom	22	*Reticulum* sp. 4	14	0.78	4	1
Tub	25	Tube sp. 23	12	0.67	3	0
Tub	25	Tube sp. 27	12	0.67	2	1
Ch-l	25	Chain sp. 7	12	0.67	3	1
Tub	28	Tube sp. 19	11	0.61	4	0
Tub	28	Tube sp. 36	11	0.61	2	0
Ch-l	28	Chain sp. 3	11	0.61	2	1
Ch-l	28	Chain sp. 12	11	0.61	1	0
		**Tot. Top 30**	**1399**	**77.8**		
		**Total 5 stations**	**1798**			

Supplementary Material S1: Description of the top-ranked species among complete specimens and fragments, live and dead combined. We follow the higher-level classification of Pawlowski et al. (2013). '*' indicates species appearing in both top-ranked categories (i.e. based on complete specimens only and fragments only).

Multichambered taxa

Tubothalamea

Ammodiscids

Usbekistania charoides (Jones & Parker) = *Glomospira charoides* Jones & Parker, 1860
See Jones (1994): Pl. 38, Figs. 13–16

Globothalamea

Rotaliids

Nuttallides umbonifera (Cushman) = *Pulvinulina umbonifera* Cushman, 1933
Supplementary Figure S2: a

Hormosinids

Hormosinella guttifera (Brady) = *Lituola guttifera* Brady, 1881
See Jones (1994): Pl. 31, Figs. 10–15

Reophax aff. *helenae* Rhumbler, 1931
Supplementary Figure S2: b, c
Test slender, fusiform, widest in the distal half, composed of 5 to 6 chambers not clearly differentiated on the test exterior, arranged along a more or less straight axis and increasing in size towards the elongate final chamber, which is drawn out into a cylindrical apertural neck. Wall thin, composed of coarse mineral grains and noticeably granular under a stereo microscope. Outer surface rather rough with fine adhering 'fluffy' particles. Length 400–800 µm.
Remarks. This species resembles *R. helenae* (Rhumbler, 1931) of Enge et al. (2012; Pl. 1, Fig. 21) from the NE Pacific (3,953 m) and Schröder (1986) from the NW Atlantic in its general form. However, according to the original description (Rhumbler, 1931), based on material collected at >4,000 m depth off St Vincent in the Caribbean, *R. helenae* has a test composed of large planktonic foraminiferal shell fragments, very different from the wall structure of the present species, hence the 'affinis' designation.

Reophax scorpiurus de Montfort, 1808
Supplementary Figure S2: d, e
Our specimens have the typical asymmetrical shape of this well-known and widely-distributed 'species', which was first described from beach sands in the Adriatic (Brönnimann and Whittaker, 1980). They resemble the specimen illustrated by Resig (1981; Pl. 9, Fig. 14). This 'species' is best considered a morphotype or species complex (Gooday and Jorissen, 2012).

Reophax sp. 1
Supplementary Figure S2: f–i
Test composed of 3–5 rounded to ovate, clearly-defined chambers, arranged along a straight or curved axis. Test coarse-grained and consisting of mineral grains. Aperture at end of short neck. Length 550–1,350 µm.
Remarks. This is one of the most common species of *Reophax* in the ABYSSLINE material. It displays some variation in the shape of the chambers, which are more elongate in some specimens than in others.

Reophax sp. 2
Supplementary Figure S2: j–l
Test composed of 2 to 3 rounded to slightly ovate chambers arranged along a more or less linear axis. Wall thin with a relatively smooth outer surface and often incorporating coloured mineral particles. Some specimens with a dusting of fine-grained particles that obscure parts of the test. The most characteristic feature is the development of a fairly long apertural neck, flared at the distal end. Length 300–800 μm.

Various MAF

?*Verneuilinulla propinqua* (Brady) = *Verneuilina propinqua* Brady, 1884
See Jones (1994): Pl.. 47, Figs. 8–12
Supplementary Figure S2: m–o
The test is large (up to ~1 mm maximum dimension), orange in colour with a fairly coarsely agglutinated wall and a fairly rough outer surface. Three chambers are more or less clearly visible in the final whorl (i.e. in end-on view) and there is a small, rounded aperture at the base of the final chamber. In the largest specimens, the test forms a rather poorly-defined spire.
Remarks. We place our specimens in this species with some hesitation. The chambers are much less clearly defined compared to Brady's original illustrations (Brady, 1884), in many cases it is difficult to discern their arrangement or the sutures between them. The test often seems quite compressed without the well-defined blunt spire seen on the original material.

Monothalamids

Monothalamids encompass a heterogeneous assortment of basically single-chambered foraminifera, which we divide into categories based mainly on morphological criteria. We regard the Komokiacea as monothalamids, although it should be noted that this group has failed to yield convincing DNA sequences (Lecroq et al., 2009).

***Nodellum*-like**

Nodellum sp. 1
Supplementary Figure S3: g
Test elongate, rigid, organic, yellowish. Slender, spindle-shaped proloculus with closed pointed proximal end. Main part of test tubular (~40–50 μm maximum width), straight or gently arcuate. One or two internal partitions may be present. Aperture terminal, round, constricted, sometimes with delicate, tubular extension. Length ~1,100–1,800 μm.
Remarks. This specie resembles *Nodellum aculeata* of (Gooday et al., 2008) from the Challenger Deep (10,896 m). However, according to the original description, *N. aculeata* is <500 μm long, much smaller than the present species, and the ratio of test width to length is 5–9%, higher than that of the present species (~2.5–4%). The difference of ~6,800 m in water depth between the Challenger Deep and UK-1 area make it unlikely that it represents the same species. Internal partitions are often present in species of *Nodellum.*

Lagenamminids and Flasks

This informal grouping includes the well-known genus *Lagenammina* and various flask-shaped morphotypes that are probably unrelated to this genus.

Lagenammina difflugiformis (Brady) = *Reophax difflugiformis* Brady, 1879
Supplementary Figure S3: b, c
Droplet-shaped chamber tapering to short apertural neck. Test wall often distinctly rusty-brown in colour, composed of fairly coarse-grained mineral particles. Length ~300–400 μm.

Remarks. Our specimens are similar to some of those assigned by Brady (1884; Pl.30, Figs. 1–4) to this species. This is probably the same species as the 'morphotype resembling *L. difflugiformis*' from the abyssal NE Atlantic, illustrated by Gooday et al. (2010; Fig. 13C) and *Lagenammina* sp. of Gooday and Jorissen (2012; Fig.2e,f). It is one of the most common species in the ABYSSLINE material.

Flask sp. 3
Supplementary Figure S3: d–f
Asymmetrical flask-shaped agglutinated chamber, ~150 µm in maximum width and 250 µm in length, including the long (~100–150 µm) tapered apertural neck. The interior is occupied by stercomata and stained material. The wall is composed of an organic layer overlain by a sparse agglutinated veneer comprising a mixture of mineral particles of different sizes, and in most cases with one or more radiolarian tests projecting from the outer surface.
Remarks. The asymmetrical shape, predominately organic test wall and presence of stercomata dissuade us from assigning this common species to the genus *Lagenammina.*

Flask sp. 4
Figure 4: d–f
Fairly coarsely-grained agglutinated chamber (~100–150 µm width), more or less spherical to somewhat angular. Two more or less straight open-ended tubes, often of different lengths (~50–100 µm and ~200–250 µm long), extend from opposite sides of the chamber. Most specimens have dead radiolarian tests attached to the outer test surface. The cytoplasm appears dark brown in colour due to the presence of stercomata.
Remarks. Morphologically, this species somewhat resembles the genus *Astrammina* in having a central chamber that gives rise to tubular extensions. However, it differs in many respects from the type species (*Astrammina rara* Rhumbler, 1932), notably its much smaller size and absence of an 'allogromiid incognito' type of cellular organisation (Bowser et al., 1995; DeLaca, 1986). *Armorella* is another similar genus but again it is larger than Flask sp. 4 with a thin, smooth test wall, a variable number of tubular processes (Höglund, 1947). Both of these genera also lack stercomata.

Spheres

Psammosphaerid sp. 19
Figure 4: j, k
Spherical agglutinated test without visible aperture, and ranging from ~100 to ~300 µm. Wall finely granular and relatively thick, with variable numbers of radiolarian tests and mineral particles attached to the outer surface. Test interior with stained cytoplasm and stercomata.

Psammosphaerid sp. 20
Supplementary Figure S3: a
Tiny (<100 µm) agglutinated sphere without visible aperture. Wall fairly thick and finely granular with a rough surface. Interior with stercomata. Often attached to bigger mineral (micronodule) or biogenic (diatom frustule, radiolarian test) particle.

Tubes

Bathysiphon sp. 2
Supplementary Figure S3: h, i
Gently tapered tubular test, up to 2.3 mm long and ~30–40 µm wide, open at both ends and following a curved or sinuous course. Surface smooth, finely agglutinated, white with a reflective sheen.
Remarks. Relatively small, white *Bathysiphon* tubes are common in deep-sea samples. Several species (e.g. *B. minutus* Pearcey, 1900 and *B. flexilis* Höglund,1947) have been described from

shallower depth, e.g. down to 700 m in the Skagerak (Höglund, 1947). However, this abyssal species is probably undescribed.

Rhizammina sp. 1
Supplementary Figure S4: g–i
Tubular test up to 1 mm or more in length and ~150–200 µm wide, straight or slightly bent, occasionally branching. Wall thick (~50 µm), comprising a mixture of fine particles, sponge spicules and subordinate numbers of radiolarian tests resulting in a very irregular outer surface. Test interior with cytoplasm forming a narrow string (~20 µm width) extending along the entire length of the tube. A somewhat wider (~40 µm width) strand of stercomata enclosed within a very delicate organic membrane runs along the tube parallel to the cytoplasm.
Remarks. The internal organisation of this species, with a strand of cytoplasm running parallel to a mass of stercomata, is typical of the genus *Rhizammina* (Cartwright et al., 1989).

Tube sp. 9
Supplementary Figure S6: a–c
Basically tubular test, up to at least 3 mm long and of variable width (~100–150 µm), with short side branches and lateral swellings. There is no visible aperture. Wall thin, basically organic with dusting of fine particles on the outer surface; conical tubercles giving rise to fine organic filaments and tubes are often developed. Test interior filled with a dense mixture of cytoplasm and stercomata.

Tube sp. 10
Supplementary Figure S5: a–c
Narrow (~50 µm width), curved to slightly sinuous agglutinated tube, up to 1 mm long. Most specimens are fragments but a closed end is present in some cases. Wall fairly thin, composed of coarse, angular grains, giving a rough 'sparkly' surface under the stereomicroscope. Cytoplasm with stercomata is visible through wall in transmitted light.

Tube sp. 19
Supplementary Figure S5: l, m
Transparent, organic-walled tube, 0.6–1.0 mm long and ~50–80 µm wide; the wall is yellow, fairly thick, sometimes with a dusting of fine-grained particles. One end of the tube may be closed. Cytoplasm filled with dense stercomata.

Tube sp. 23
Supplementary Figure S6: d–f
Delicate, branching, basically tubular test, ~50–80 µm wide and a few 100 µm in length. Wall composed of an organic layer covered by a thin dusting of fine-grained particles, and often incorporating radiolarian tests and large mineral grains. The organic layer gives rise to numerous short filaments extending from the organic part of the wall, giving a very fuzzy, ragged aspect. Cytoplasm contains large but relatively sparse stercomata.

Tube sp. 25
Supplementary Figure S5: f–i
Relatively short, more or less straight, slightly tapered tubular test, ~400–1,000 µm long and ~100 µm wide. One end is typically closed and the other end open. Wall noticeably granular under stereomicroscope, composed of small, angular particles and incorporating variable numbers of much larger radiolarian tests. Interior with stercomata embedded in red-stained material.

Tube sp. 27
Supplementary Figure S5: j, k
Small, slender, spindle-shaped to tubular test, <500 µm long and ~40 µm maximum width. Wall with yellowish tinge and a variably developed, finely-agglutinated veneer overlying an organic base; a few scattered larger particles are sometimes present. Test contents finely granular, containing numerous tiny stercomata-like bodies.

Tube sp. 32*
Supplementary Figure S3: j–l
Basically tubular test, from a few 100 µm up to 2.6 mm long and ~150–250 µm wide, usually more or less straight or slightly crooked, sometimes with slight constrictions or swollen sections. One or both ends are closed in specimens that appear complete. Wall fairly thin but semi-opaque, finely agglutinated and typically with tiny scattered dark particles. A few larger biogenic particles and micronodules may be attached on the wall.
Remarks. Smaller specimens of this fairly distinctive species are more equidimensional than tubular, making it difficult to classify this species morphologically. It somewhat resembles the xenophyophore genus *Aschemonella* but lacks the internal features (stercomare and granellare) typical of xenophyophores.

Tube sp. 36
Supplementary Figure S6: g–h
Delicate, fragmentary, tubular test, unbranched and either straight or gently curved; fragment are <500 µm long and ~100 µm wide. Wall with a distinct basal organic layer overlain by a very heterogeneous and coarse-grained mixture of angular quartz grains and biogenic particles, mainly radiolarian tests, that project to create a very uneven outer surface. Cytoplasm filled with stercomata.

Tube sp. 40*
Supplementary Figure S5: d, e
Unbranched, tubular test, typically fragmented, 500 µm or more long and of somewhat variable width (generally ~150–200 µm), agglutinated with coarse, angular particles, with one closed end and an aperture at the opposite end. The tube follows a more or less straight or somewhat crooked course. Wall is noticeably granular under the stereomicroscope and composed of small quartz grains. It may give rise to fine, flexible filaments, although these are not always developed. Cytoplasm contains sparse stercomata.

Tube sp. 41
Supplementary Figure S6: i, j
Test forms a curved, sometimes slightly twisted, tapered tube, ranging in length from ~0.5 to 2.3 mm (measured along the length) and with a maximum width of ~150 µm. Transverse constrictions are more or less well developed and the narrower (proximal) end is closed, forming a rounded proloculus (40–80 µm diameter). The test interior is interrupted by occasional transverse septae. The entire tube is reddish in colour when stained. The wall is translucent and comprises a relatively thick organic base overlain by a fine-grained agglutinated layer of variable thickness. Cytoplasm contains stercomata.

Tube sp. 43
Figure 5: n–p
Delicate, brownish tubular test that is occasionally branched. The longest pieces are 500–900 µm in length and ~80–100 µm wide. Specimens are often open-ended and appear to be fragments, but one closed end is sometimes observed. The tubes have a neat, finely agglutinated wall, ~20 µm thick, and at least one closed end. The test interior contains dark stercomata that appear, either singly or in clumps, as a series of dots along the length of the tube.

Tube sp. 44*
Supplementary Figure S7: i, j
Delicate, narrow, branching tube, typically ~0.5 up to 1.5 mm long and <50 µm wide. The wall is thin and composed of fine particles. There is no obvious aperture, although the ends of the tubes are often broken. Cytoplasm contains numerous stercomata.
Remarks. Resembles Tube sp. 43 but is considerably narrower and more often branched.

Tube sp. 45
Supplementary Figure S7: a–f
Tubular test, ~0.6–1.4mm long (complete specimens) and~100–150 µm wide, either branched or unbranched, sometimes with short side lobes; ends are sometimes closed. Test wall soft and fine-grained and of relatively even thickness (~20 µm). Test interior with dense stercomata.
Remarks. Resembles Tube sp. 43 but the tubular test is distinctly wider.

Tube sp. 46
Supplementary Figure S7: g, h
Tubular test follows a more or less straight or somewhat crooked course; it is usually unbranched but sometimes has a short side lobe. Intact ends are closed, but all available specimens appear to be fragments with one or both ends broken. Most are <1 mm but the longest reaches ~3 mm in length; the width varies from 230 to 350 µm. The wall is thick, finely agglutinated with a slightly fuzzy surface; the thickness is generally between 25 and 35 µm but a thinner-walled variant also occurs. The test interior is densely packed with a mass of stercomata and red-stained material, the outline of which is visible through the wall when the tube is immersed in glycerol.

Tube sp. 48
Figure 4: a–c
Typical specimens are elongate and best described as approximately spindle-shaped, tapering towards both ends and sometimes with short triangular processes or tapering side branches. These typical spindles grade into more tubular tests. Most specimens are probably complete and measure from ~0.3 to 0.8 mm long and 60 to 220 µm wide. Occasionally, two spindles are joined end to end. The wall is translucent with a very fine granularity visible when viewed under the stereomicroscope. Under high magnification in a compound microscope, the wall appears basically organic with a variably developed veneer of fine particles, normally 5–15 µm thick but sometimes more. In some samples, a few radiolarians or other biogenic particles project from the test wall. Large, dark stercomata, sometimes densely packed, are a prominent feature of the test interior. Several variants are included within this form. In one type, the test has a smoother outline, is devoid of radiolarians, triangular processes and short side branches but sometimes bifurcates. Another variant has a rather irregular shape and a 'fluffy' surface with short filaments. All types contain dense stercomata.
Remarks. The combination of these variants as Tube sp. 48 represents a compromise between 'lumping' and 'splitting' based on a thorough examination of all available material. It is possible, however, that this morphospecies encompasses several genetically distinct species.

Tube sp. 54*
Figure 4: g–i
Test basically tubular to spindle-shaped, 0.2–0.8 mm long and relatively wide (~50–150 µm); some specimens bifurcate while others comprise two spindle-shaped elements, one of which buds off the side of the other. Wall fairly delicate and seems basically organic with variably developed surface layer of flat-lying, mainly siliceous particles resulting is a relatively smooth outer surface. Test contents very heterogeneous, including stercomata but also a variety of crumpled organic sheets and organic-walled cyst-like structures. A variant form includes tubular to spindle-shaped tests of similar size to typical specimens. Some intact tests have one end that is rounded and closed and the

other end narrower and drawn out into a delicate organic filament. Compared to typical specimens, this form has a thicker wall that is more distinctly granular when observed in a compound microscope. The test interior is also dominated to a much greater extent by dark stercomata.
Remarks. We originally regarded these two forms as separate species. However, careful examination revealed a continuum between specimens with coarse test walls and those with few agglutinated particles as well as variability in the test contents.

Tube sp. 56
Supplementary Figure S4: a–c
Tubular test following a straight, curved to slightly bent or crooked course. Specimens range from 1 mm to 3 mm long and ~100–150 µm wide. Most have open ends and are probably fragments, but occasional pieces have one closed end. Most of the available fragments are unbranched but the bases of branches are sometimes present. The wall is well-defined, relatively thick, fine-grained with a smooth outer surface. The test interior is almost completely filled with a dense mass of stercomata and red-stained bodies.
Remarks. This species was originally assigned to *Rhizammina*, which it resembles in overall form. However, the presence of tubes with closed ends, and the lack of a distinct strand of cytoplasm running longitudinally along the tube parallel to a mass of stercomata, are features that seem incompatible with a placement in this genus (Cartwright et al., 1989). Tube sp. 56 is rather similar to *Aschemonella tubulosa* Kamenskaya, Gooday, Tendal 2016 (Kamenskaya et al., 2016), but the former species is somewhat narrower and lacks the rounded stercomata masses present in *A. tubulosa.*

Tube sp. 57
Supplementary Figure S4: d–f
Unbranched or occasionally branched tubular test, up to 1 cm or more in length and 75–100 µm wide, open at both ends and following a curved, often somewhat crooked course. Wall finely granular with relatively smooth outer surface. Interior with dense stercomata and red-stained material.
Remarks. Tube sp. 57 differs from Tube sp. 56 in being somewhat narrower and always open-ended rather than sometimes having a closed end. Although the agglutinated wall is fine-grained, the grains are noticeably larger than those that constitute the wall of Tube sp. 56 when observed under a compound microscope. Like Tube sp. 56, this species lacks internal features typical of *Rhizammina,* a genus to which it was originally assigned.

Chains

Although superficially appearing multichambered, we consider chain-like morphotypes to comprise a series of ‘pseudo-chambers’ or ‘segments’ rather than true chambers, and therefore include them in the monothalamids.

Chain sp. 3
Supplementary Figure S8: a, b
Chain-like agglutinated test with oval to droplet-shaped pseudo-chambers (length ~200 µm) joined by short, wide necks (width ~100 µm), and filled with dark cytoplasm and stercomata. Some chambers have two short, open-ended, tubular extensions, either apertural structures or the result of breakage. Wall thin, finely granular with relatively smooth outer surface.
Remarks. In terms of test morphology, this species somewhat resembles the xenophyophore genus *Aschemonella*. However, it is smaller than any known species of *Aschemonella* and lacks the distinctive internal features (stercomare and granellare) that this genus shares with other xenophyophores.

Chain sp. 7
Supplementary Figure S8: c, d
Chain-like agglutinated test comprising several relatively small droplet-shaped pseudo-chambers (width ~80–100 µm, length ~100–150 µm) joined end to end by short tapered necks and filled by dark stercomata and stained material. Wall finely granular but often incorporating much larger particles, with a visible inner organic layer.
Remarks. This species is smaller and more delicate than Chain sp. 3 and the test wall is constructed from a more heterogeneous selection of particles.

Chain sp. 12
Supplementary Figure S8: e, f
Very flimsy chain-like form, the available specimens comprising two irregular segments joined by a narrow neck. Segments vary significantly in shape within a single specimen and between specimens; they are usually elongated and sometimes with spiky protuberances. Wall thin, translucent, fine-grained and delicate. Test interior with stercomata and red-stained material.

Chain sp. 13*
Figure 5: d–g
The elongate test is 0.5–1.5 mm long in specimens that are apparently complete (although most appear to be fragments) and ranges from <100 µm to ~260 µm in width, according to the thickness of the agglutinated layer. It occasionally branches but most specimens are unbranched. The wall is basically organic so that the entire test stains red. The organic layer is overlain by a layer of finely agglutinated material that varies in thickness from 90–100 µm (in which case the test interior is largely obscured) to ~15 µm, or in some cases is almost non-existent. Beneath this layer, the basically tubular organic part of the test is partitioned into a series of globular pseudo-chambers, each containing a clump of dark stercomata.

Unclassified

Monothalamid sp. 17
Supplementary Figure S9: a–c
Test variously shaped but generally elongated and more or less oval, sometimes more lobate. Length ~0.5–1.1 mm, width ~200–300 µm. Wall thick (~30–40 µm), semi-opaque and fine-grained, sometimes with variably developed hair-like filaments ~40–150 µm in length. Test interior with dense cytoplasm containing stercomata.

Monothalamid sp. 19
Supplementary Figure S9: d–f
Test rounded, generally ovate, sometimes more irregular in shape; small lobes may be developed, usually at one end of the test. Length ~0.20–0.45 mm. Wall translucent to almost transparent, composed of an organic inner layer covered by a very thin layer of fine-grained agglutinated material. There is no obvious aperture. Test content very heterogeneous, including stercomata together with sheet-like and cyst-like structures of probable biogenic origin.
Remarks. Although the overall morphology is different, the heterogeneous test contents of this species closely resemble those of Tube sp. 54.
Monothalamid sp. 23
Supplementary Figure S9: g, h
Test usually rounded to ovate, ~150–250 µm wide, in one case (illustrated) with two rounded pseudo-chambers arising from a central tubular feature. Wall thick (~30–40 µm), fine-grained with a 'fluffy' surface, light brown in reflected light, appearing dark brown when viewed in transmitted light. Test contents include dense mass of stercomata.
Remarks. The illustrated specimen superficially resembles a komokiacean fragment. However, the 2 rounded pseudo-chambers are larger and the wall thicker than in members of this group.

Monothalamid sp. 27*
Figure 5: h–j
Agglutinated test typically resembling a triangle or an arrow head with three or four pointed ends. Wall neat, fine-grained, ~20–30 µm thick, and sometimes slightly produced at the pointed ends where it becomes much thinner. One wider variant (Fig. 5i) divides into two short broad branches with lobate extremities tapering on the opposite side to a single aperture. Length ~200–450 µm. Width of 'pointed' variant ~200–300 µm, and width of 'branched variant ~420–600 µm.

Monothalamid sp. 32
Figure 5: a–c
Delicate, approximately tubular structure (~80–100 µm width) with poorly-defined segments, each containing a discrete oval mass of brightly stained cytoplasm dominated by spherical stercomata. Wall is poorly-defined and consists of fine-grained material overlying an inner organic membrane.
Remarks. This enigmatic form is difficult to interpret. The cytoplasmic masses appear to be unconnected, although in life they were possibly linked by cytoplasmic strands.

Monothalamid sp. 33
Supplementary Figure S9: i, j
Compact cluster of globular chambers (~60–75 µm diameter); the largest cluster, which is probably still a fragment, measures ~350 µm in maximum dimension and contains around 10 chambers. Wall ~10 µm thick and fine-grained. Chambers contain clumps of stercomata.
Remarks. The chambers resemble those of komokiaceans but there is no sign of the tubules that characterise this taxon.

Komokiacean-like

Komokiacean-like sp. 20*
Figure 4: l, m
Variably shaped test, often subtriangular, spindle-shaped or more rounded and often with tubular extensions (~40–100µm long). Komokiacean-like wall structure with a fine-grained agglutinated veneer overlying an organic layer. Test interior compartmentalised to resemble a honeycomb and filled with masses of stercomata. Maximum dimension ~150–180 µm (without tubular extensions).

Komokiacea: Baculellidae

Baculella globofera Tendal & Hessler, 1977
See Tendal and Hessler (1977): Pl. 15, Figs. A, B

Edgertonia floccula Shires, Gooday & Jones, 1994
See Shires et al. (1994): Pl. 1, Figs. 1–7

Edgertonia sp. 4
Supplementary Figure S10: a, b
Clusters of short, finger-like branching tubules, typically ~50 µm wide, usually ending in slightly bulbous swellings or clusters of swellings. Tubes and swellings contain discrete clumps of stercomata, often separated by more or less distinct septae that subdivide the interior into compartments. Wall organic with finely granular outer surface.

Edgertonia sp. 5
Supplementary Figure S10: c, d
Branching tubules (~50 µm wide) ending in 1–2 bulbous swellings, ~100 and ~200 µm maximum dimension. Wall composed of organic layer overlain by variably developed agglutinated veneer. Test interior with sparse stercomata and stained material.

Remarks. This form differs from *Edgertonia* sp. 4 in having larger and more pronounced terminal swellings, no septae, and a flimsier wall.

Edgertonia sp. 7
Figure 5: k–m
Irregularly-shaped cluster of globular chambers (each ~50 µm wide), loosely to fairly tightly packed within a variably-developed fine-grained matrix and sometimes surrounded by fine organic fibres. Individual clusters measure up to 750 µm (usually 250–500 µm) in maximum dimension.

Komokiacea: Komokiidae

Lana sp. 1
Supplementary Figure S11: e, f
Test consisting of sparsely branching, sometimes anastomosing tubules. Tubules are non-septate, parallel-sided, and the sections between the branches are more or less straight. Organic layer of the wall distinct, covered by a fairly thick coating of fine-grained particles creating a somewhat fuzzy surface. The width of the inner organic tubule = 19–24 µm, width of the outer agglutinated layer = 10–20 µm, total tubule width = 45–60 µm. All specimens were regarded as fragments.

Reticulum sp. 1
Supplementary Figure S11: a, b
Test consists of branching and anastomosing tubules. Tubules non-septate, more or less parallel-sided and following a somewhat irregular course between the branches. Distinct organic layer of wall overlain by variably developed fine-grained coating. The width of the inner organic tubule = 14–19 µm, width of the outer agglutinated layer = 5–6 µm, total tubule width = 22–30 µm.
Remarks. This form is similar to *Lana* sp. 1 but has somewhat narrower tubules, a less distinct organic wall layer and a thinner coating of fine-grained particles. Fragments are also more commonly reticulated, hence its placement in the genus *Reticulum*.

Reticulum sp. 4
Supplementary Figure S11: c, d
Loose clump of narrow, branching, reticulated tubules (~10–15 µm width). Tubules non-septate, parallel-sided. Organic wall covered by a very thin fine-grained coating. Clumps, assumed to be fragments, are up to 1 mm or more in size. We place this species in the genus *Reticulum* because the tubules are fairly tightly meshed, more so than in the closely related genus *Lana*.

Supplementary Material S2: Taxonomic list of taxa assigned to a named species that do not appear in the top-ranked species among complete specimens and fragments (live and dead combined). References to plates and figures in literature are provided.

Multichambered taxa

Tubothalamea

Miliolids

Cornuspira involvens = *Operculina involvens* Reuss, 1850
See Jones (1994): Pl. 11, Figs. 1–2

Ammodiscids

Glomospira gordialis = *Trochammina squamata* var. *gordialis* Jones & Parker, 1860
See Jones (1994): Pl. 38, Figs. 7–9

Globothalamea

Robertinids

Hoeglundina elegans = *Rotalia* (*Turbinuline*) *elegans* d'Orbigny, 1826
See Enge et al. (2012): Pl. 3, Fig. 24

Rotaliids

Alabaminella weddellensis = *Eponides weddellensis* Earland, 1936
See Earland (1936): Pl. 1, Figs. 65–67; Cornelius and Gooday (2004): Figs. 6A,C; Loeblich and Tappan (1988): Pl. 593, Figs. 18–22

Cibicidoides mundula = *Truncatulina mundula* Brady, Parker & Jones, 1888
See Jones (1994): Pl. 95, Fig. 6; Holbourn et al. (2013): page 196.

Epistominella exigua = *Pulvinalina exigua* Brady, 1884
See Holbourn et al. (2013): p. 240

Globocassidulina subglobosa = *Cassidulina subglobosa* Brady, 1881
See Jones (1994): Pl. 3, Fig. 12

Melonis pompiloides = *Anomalina pompilioides* Fichtel & Moll, 1798
See Jones (1994): Pl. 108, Figs. 10–11

Pullenia bulloides = *Nonionina bulloides* d'Orbigny, 1846
See Jones (1994): Pl. 84, Figs. 12–13

Pullenia catalinaensis McCulloch, 1977
See Setoyama and Kaminski (2015): Pl. 6, Figs 9a–b, 10a–b

Pullenia quinqueloba = *Nonionina quinqueloba* Reuss, 1851
See Jones (1994): Pl. 84, Figs. 14–15

Hormosinids

Hormosinella distans = *Lituola distans* Brady, 1881
See Schröder et al. (1988): Pl. 5, Fig. 7 (as *Reophax distans*)
Hormosinella ovicula = *Hormosina ovicula* Brady, 1879
See Jones (1994): Pl. 39, Figs. 7–9

Nodosinum gaussicum = *Nodosinella gaussica* Rhumbler, 1913
See Jones (1994): Pl. 31, Figs. 1–2, 5 (as *Reophax gaussicus*)

Reophax sp. 112/113 in Gooday et al. (2010): Fig. 14A
See Stefanoudis et al. (2016): Pl. 1, Figs. 11–12 (as *Reophax* sp. 9)

Trochamminids

Deuterammina grahami Brönniman & Whittaker, 1988
See Brönnimann and Whittaker (1988): Figs. 38A–J, 39A–C

Paratrochammina challengeri Brönniman & Whittaker, 1988
See (Brönnimann and Whittaker, 1988): Figs. 16H–K

Various MAF

Adercotryma glomerata = *Lituola glomerata* Brady, 1878
See Jones (1994): Pl. 34, Figs. 15–18

Ammomarginulina recurva Earland, 1934
See Cornelius and Gooday (2004): Fig. 4O; Enge et al. (2012): Pl. 1, Fig. 26 (as *Eratidus foliaceus recurvus*).

Buzasina galeata = *Trochammina galeata* Brady, 1881
See Jones (1994): Pl. 40, Figs. 19–23

Cribrostomoides subglobosa (Cushman) = *Alveolophragmium subglobosum* Cushman, 1910
See Jones (1994): Pl. 34, Figs. 8–10

Cyclammina trullissata = *Trochammina trullissata* Brady, 1879
See Jones (1994): Pl. 40, Fig. 6

Cystammina pauciloculata = *Cyclammina pauciloculata* Brady, 1879
See Jones (1994): Pl. 41, Fig. 1

Eggerella bradyi = *Verneuilina bradyi* Cushman, 1911
See Enge et al. (2012), Pl. 2, Fig. 5

Veleroninoides scitula = *Lituola* (*Haplophragmium*) *scitulum* Brady, 1881
See Jones (1994): Pl. 34, Fig. 11–13

Veleroninoides wiesneri = *Labrospira wiesneri* Parr, 1950
See Jones (1994): Pl. 40, Fig. 14

Monothalamids

Nodellum-like

Resigella moniliforme = *Nodellum moniliforme* Resig, 1982
See Gooday et al. (2008): Figs. 9–10

Lagenamminids

Lagenammina tabulata Rhumbler, 1931
See Enge et al. (2012): Pl. 1, Fig. 8

Organic-walled

Tinogullmia riemanni Gooday, 1990
See Gooday (1990): Figs. 3, 7

Spheres

Crithionina hispida = *Crithionina pisum* var. *hispida* Flint, 1899
See Flint (1899): Pl. 6, Fig. 2; Höglund (1947): Pl. 2, Fig. 3

Thurammina papillata Brady, 1879
See Jones (1994): Pl. 36, Fig. 7

Hyperamminids

Hyperammina cylindrica Parr, 1950
See Jones (1994): Pl. 23, Fig. 7

Tubes

Saccorhiza ramosa = *Hyperammina ramosa* Brady, 1879
See Jones (1994): Pl. 23, Figs. 15–19

Supplementary Material S3: List of references cited in Supplementary Table S1 and Supplementary Materials S1 and S2.

Bowser, S.S., Gooday, A.J., Alexander, S.P., Bernhard, J.M., 1995. Larger agglutinated foraminifera of McMurdo Sound, Antarctica: Are *Astrammina rara* and *Notodendrodes antarctikos* allogromiids incognito? *Mar. Micropaleontol.* 26, 75–88.

Brady, H.B., 1884. *Report on the foraminifer dredged by H.M.S. Challenger during the years 1873–1876. Report of the scientific results of the voyage of H.M.S. Challenger, 1873–1876.* Zoology 9, London, England.

Brönnimann, P., Whittaker, J.E., 1988. *Trochamminacea of the Discovery reports.* British Museum (Natural History).

Brönnimann, P., Whittaker, J.E., 1980. *A Revision of Reophax and Its Type-species, with Remarks on Several Other Recent Hormosinid Species (Protozoa: Foraminiferida) in the Collections of the British Museum (Natural History).* British Museum (Natural History).

Cartwright, N.G., Gooday, A.J., Jones, A.R., 1989. The morphology, internal organization, and taxonomic position of *Rhizammina algaeformis* Brady, a large, agglutinated, deep-sea foraminifer. *J. Foraminifer. Res.* 19, 115–125.

Cornelius, N., Gooday, A.J., 2004. "Live" (stained) deep-sea benthic foraminiferans in the western Weddell Sea: trends in abundance, diversity and taxonomic composition along a depth transect. *Deep-Sea Res. II* 51, 1571–1602.

DeLaca, T.E., 1986. The morphology and ecology of *Astrammina rara. J. Foraminifer. Res.* 16, 216–223.

Earland, A., 1936. Foraminifera, Part IV. Additional records from the Weddell Sea sector from material obtained by the S.Y. "Scotia". *Discov. Rep.* 13, 1–76.

Enge, A.J., Kucera, M., Heinz, P., 2012. Diversity and microhabitats of living benthic foraminifera in the abyssal Northeast Pacific. *Mar. Micropaleontol.* 96, 84–104.

Flint, J.M., 1899. *Recent Foraminifera: A descriptive catalogue of specimens dredged by the US Fish Commission Steamer Albatross.* Smithsonian Institution, United States National Museum.

Gooday, A.J., 1990. *Tinogullmia riemanni* sp. nov. (Allogromiina; Foraminiferida), a new species associated with organic detritus in the deep sea. *Bull. Br. Mus. Nat. Hist. Zool.* 56, 93–103.

Gooday, A.J., Jorissen, F.J., 2012. Benthic Foraminiferal Biogeography: Controls on Global Distribution Patterns in Deep-Water Settings. *Annu. Rev. Mar. Sci.* 4, 237–262, doi:10.1146/annurev-marine-120709-142737.

Gooday, A.J., Malzone, M.G., Bett, B.J., Lamont, P.A., 2010. Decadal-scale changes in shallow-infaunal foraminiferal assemblages at the Porcupine Abyssal Plain, NE Atlantic. *Deep-Sea Res. II* 57, 1362–1382, doi:10.1016/j.dsr2.2010.01.012.

Gooday, A.J., Todo, Y., Uematsu, K., Kitazato, H., 2008. New organic-walled Foraminifera (Protista) from the ocean's deepest point, the Challenger Deep (western Pacific Ocean). *Zool. J. Linn. Soc.* 153, 399–423, doi:10.1111/j.1096-3642.2008.00393.x.

Höglund, H., 1947. *Foraminifera in the Gullman Fjord and the Skagerak.* Zoologiska Birdrag från Uppsala XXVI, 49–50, 531 p.

Holbourn, A., Henderson, A.S., MacLeod, N., 2013. *Atlas of benthic foraminifera.* John Wiley & Sons.

Jones, R.W., 1994. *The Challenger foraminifera.* Oxford University Press and The Natural History Museum, London.

Kamenskaya, O.E., Gooday, A.J., Tendal, O.S., Melnik, V.F., 2016. Xenophyophores (Rhizaria, Foraminifera) from the Russian license area of the Clarion–Clipperton Zone (eastern equatorial Pacific), with the description of three new species. Mar. Biodivers. 1–8, doi:10.1007/s12526-016-0595-x.

Lecroq, B., Gooday, A.J., Cedhagen, T., Sabbatini, A., Pawlowski, J., 2009. Molecular analyses reveal high levels of eukaryotic richness associated with enigmatic deep-sea protists (Komokiacea). *Mar. Biodivers.* 39, 45–55.

Loeblich, A.R., Tappan, H., 1988. *Foraminiferal genera and their classification.* Van Nostrand Reinhold, New York.

Pawlowski, J., Holzmann, M., Tyszka, J., 2013. New supraordinal classification of Foraminifera: Molecules meet morphology. *Mar. Micropaleontol.* 100, 1–10.

Resig, J.M., 1981. Biogeography of benthic foraminifera of the northern Nazca plate and adjacent continental margin. *Geol. Soc. Am. Mem.* 154, 619–666, doi:10.1130/MEM154-p619.

Schröder, C.J., 1986. Deep-water arenaceous foraminifera in the northwest Atlantic Ocean. *Can. Tech. Rep. Hydrogr. Ocean Sci.* 71, 1–191.

Schröder, C.J., Scott, D.B., Medioli, F.S., Bernstein, B.B., Hessler, R.R., 1988. Larger agglutinated foraminifera; comparison of assemblages from central North Pacific and western North Atlantic (Nares Abyssal Plain). *J. Foraminifer. Res.* 18, 25–41, doi:10.2113/gsjfr.18.1.25.

Setoyama, E., Kaminski, M.A., 2015. Neogene benthic foraminifera from the southern Bering Sea (IODP Expedition 323). *Palaeontol. Electron.* 18, 1–30.

Shires, R., Gooday, A.J., Jones, A.R., 1994. The morphology and ecology of an abundant new komokiacean mudball (Komokiacea, Foraminiferida) from the bathyal and abyssal NE Atlantic. *J. Foraminifer. Res.* 24, 214–225.

Stefanoudis, P.V., Schiebel, R., Mallet, R., Durden, J.M., Bett, B.J., Gooday, A.J., 2016. Agglutination of benthic foraminifera in relation to mesoscale bathymetric features in the abyssal NE Atlantic (Porcupine Abyssal Plain). *Mar. Micropaleontol.* 123, 15–28.

Tendal, O.S., Hessler, R.R., 1977. An introduction to the biology and systematics of Komokiacea. *Galathea Rep.* 14, 165–194.

Supplementary Figure S1: Species ranked by abundance based on complete (live and dead) specimens (all samples combined). For information, the level of 5 specimens is indicated by a dotted line.

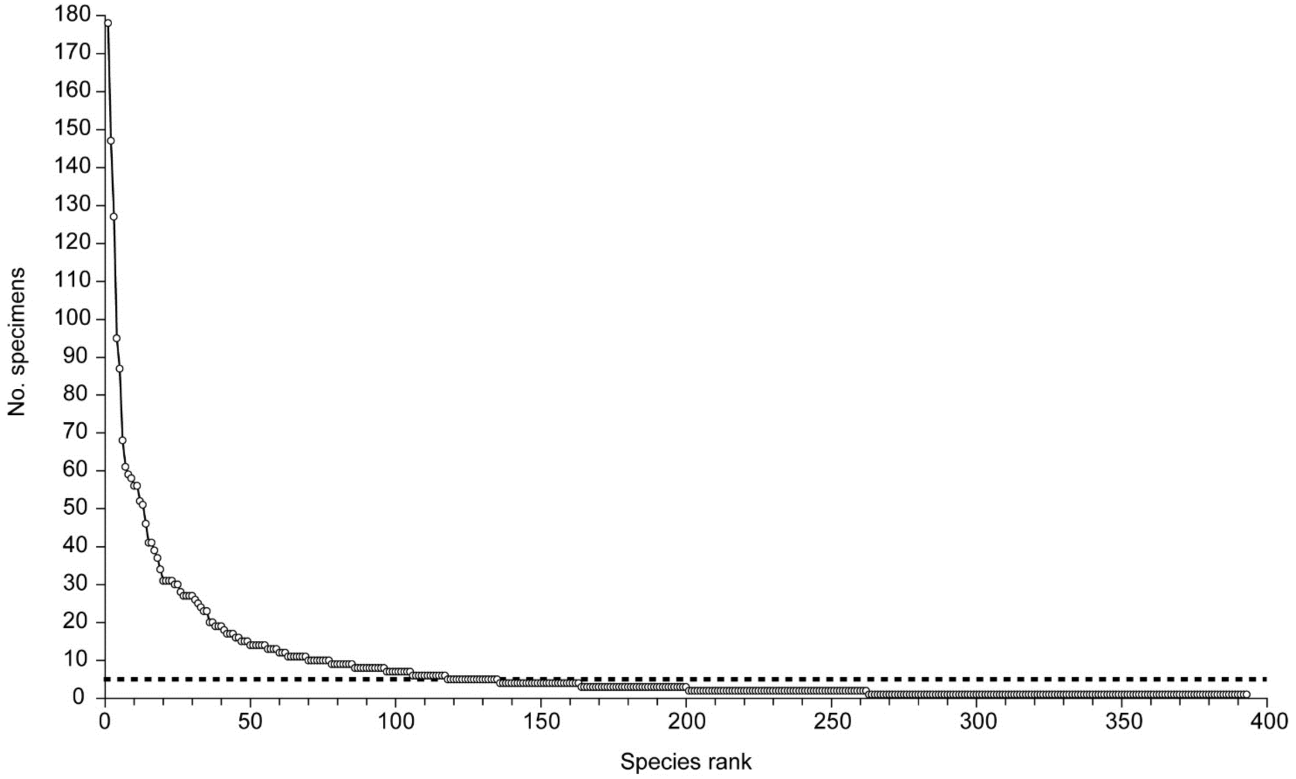

Supplementary Figure S2: Reflected (a, b, d, f–l) and transmitted (c, e) light photographs of 5 of the 9 most abundant multichambered species represented by complete specimens or fragments (live and dead combined). (a) *Nuttalides umbonifera*. (b, c) *Reophax* aff. *helenae* (same specimen). (d, e) *Reophax scorpiurus* (same specimen). (f–i) *Reophax* sp.1 (4 specimens). (j–l) *Reophax* sp.2 (3 specimens). (m–o) ?*Verneuilinulla propinqua* (3 specimens). All scale bars 100 µm except where indicated otherwise.

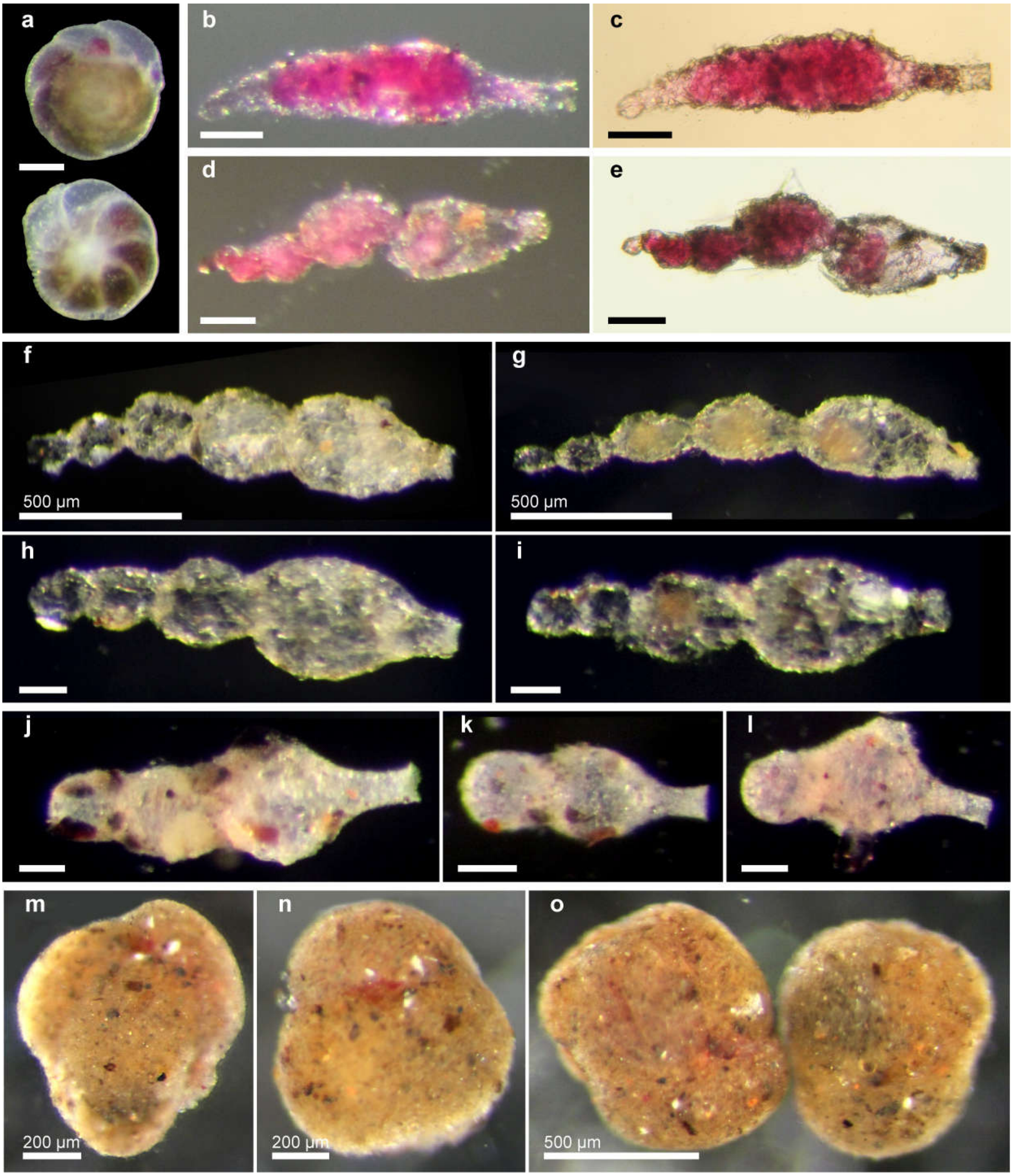

Supplementary Figure S3: Reflected (b, d, e, h, j) and transmitted (a, c, f, g, i, k, l) light photographs of 6 of the 37 most abundant other monothalamous species represented by complete specimens or fragments (live and dead combined). (a) *Psammosphaerid* sp. 20. (b, c) *Lagenammina difflugiformis* (2 specimens). (d–f) Flask sp. 3; overview of 4 specimens (d) and detail of one specimen (e, f). (g) *Nodellum* sp. 1. (h, i) *Bathysiphon* sp. 2; overview (h) and detail (i) of same specimen. (j–l) Tube sp. 32; overview of one specimen (j) and detail of another specimen (k, l) small black mineral grains incorporated into the wall (k) and the test interior enclosing stained material and stercomata (l). All scale bars 100 µm except where indicated otherwise.

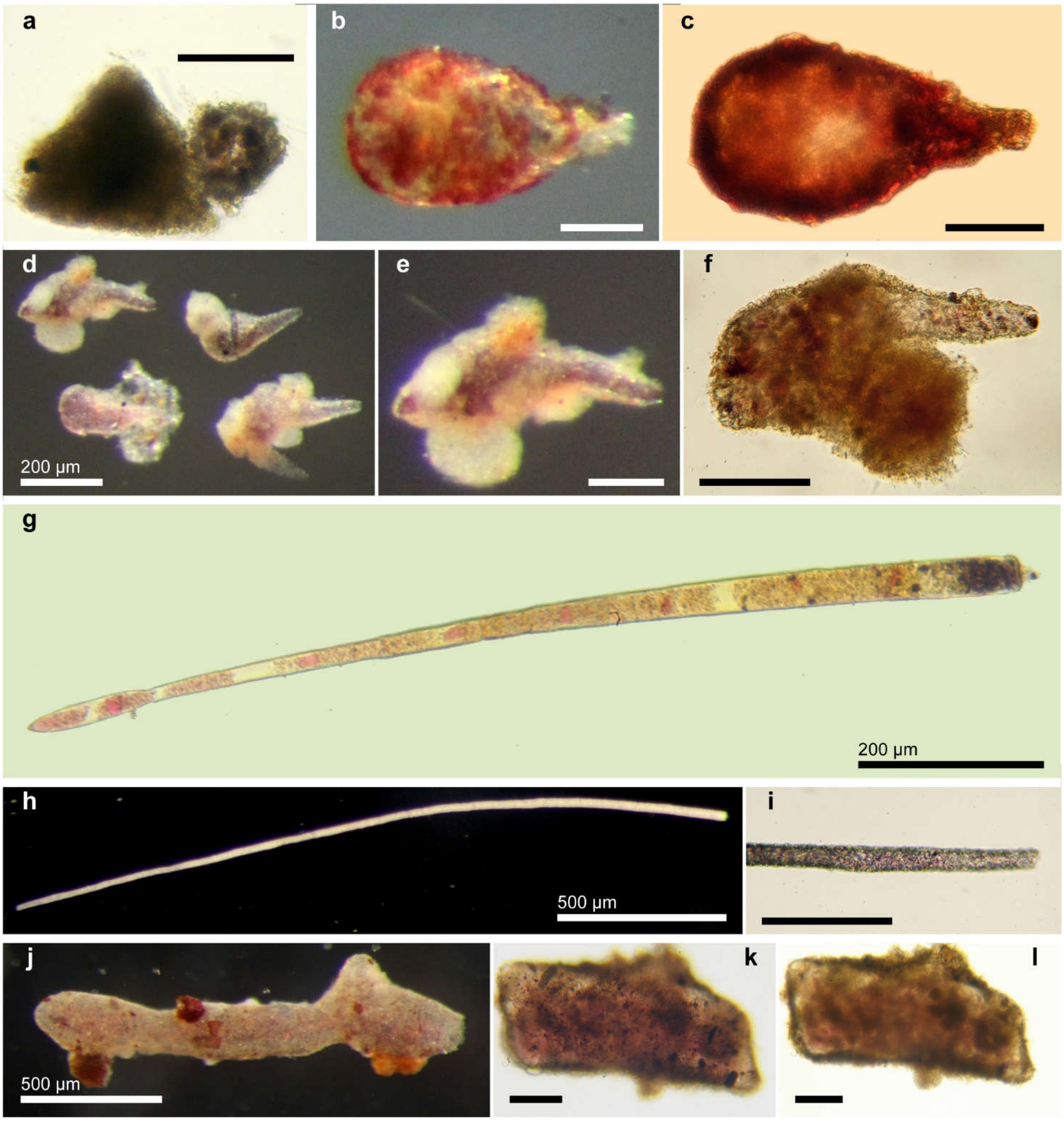

Supplementary Figure S4: Reflected (a, b, d, e, g) and transmitted (c, f, h, i) light photographs of 3 of the 37 most abundant other monothalamous species represented by complete specimens or fragments (live and dead combined). (a–c) Tube sp. 56; overview of 2 specimens (a), a third specimen (b) and detail of third specimen (c). (d–f) Tube sp. 57; overview (d) and detail (e, f) of same specimen. (g–i) *Rhizammina* sp. 1; overview (g, h) and detail (i) of same specimen showing stercomata mass (brownish structure) and the Rose Bengal-stained string of cytoplasm. All scale bars 100 µm except where indicated otherwise.

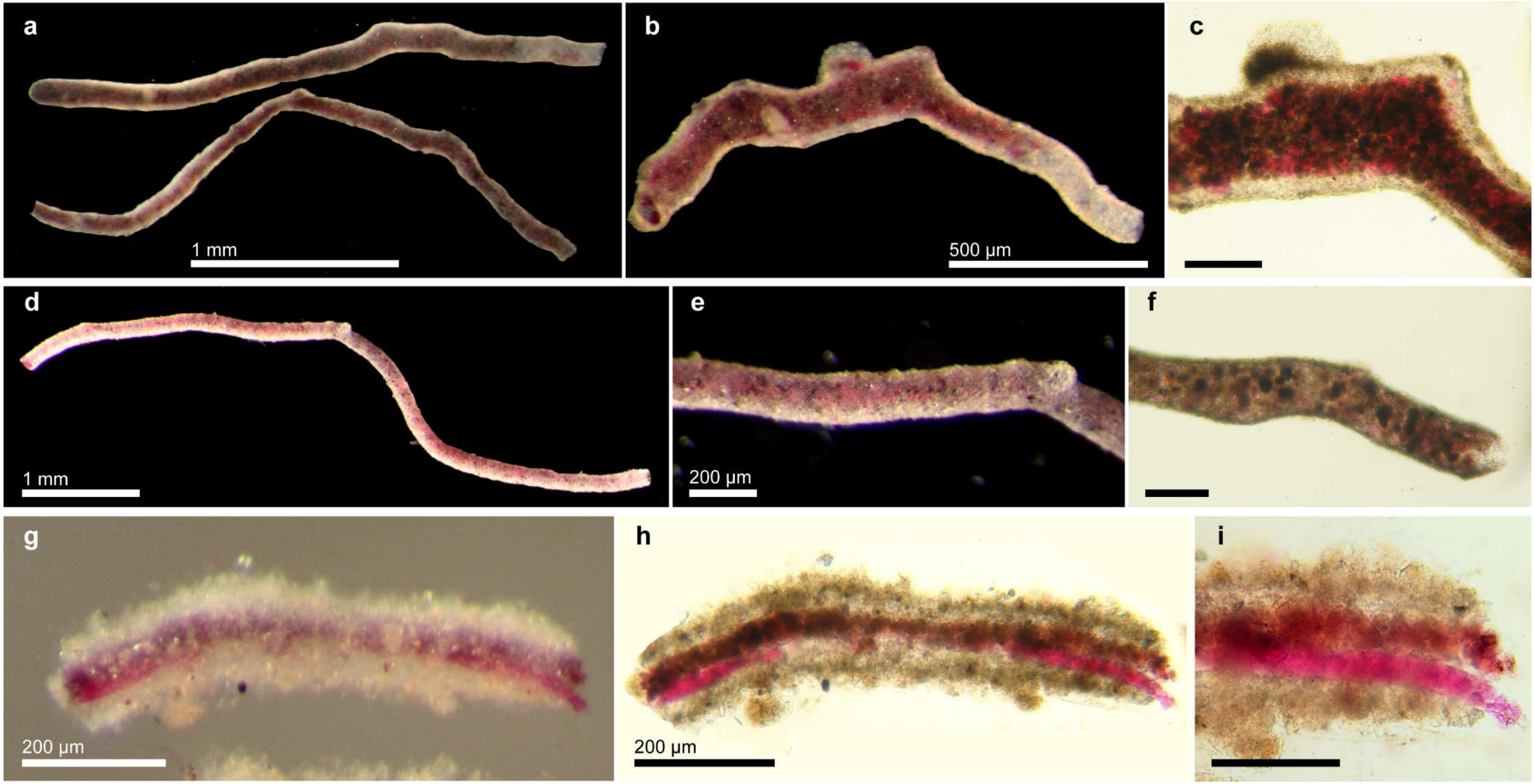

Supplementary Figure S5: Reflected (a, d, f, g, j, l) and transmitted (b, c, e, h, i, k, m) light photographs of 4 of the 37 most abundant other monothalamous species represented by complete specimens or fragments (live and dead combined). (a–c) Tube sp. 10; overview (a, b) and detail (c) of same specimen. (d, e) Tube sp. 40 (same specimen). (f–i) Tube sp. 25 (4 specimens). (j, k) Tube sp. 27 (same specimen). (l, m) Tube sp. 19 (2 specimens). All scale bars 100 µm except where indicated otherwise.

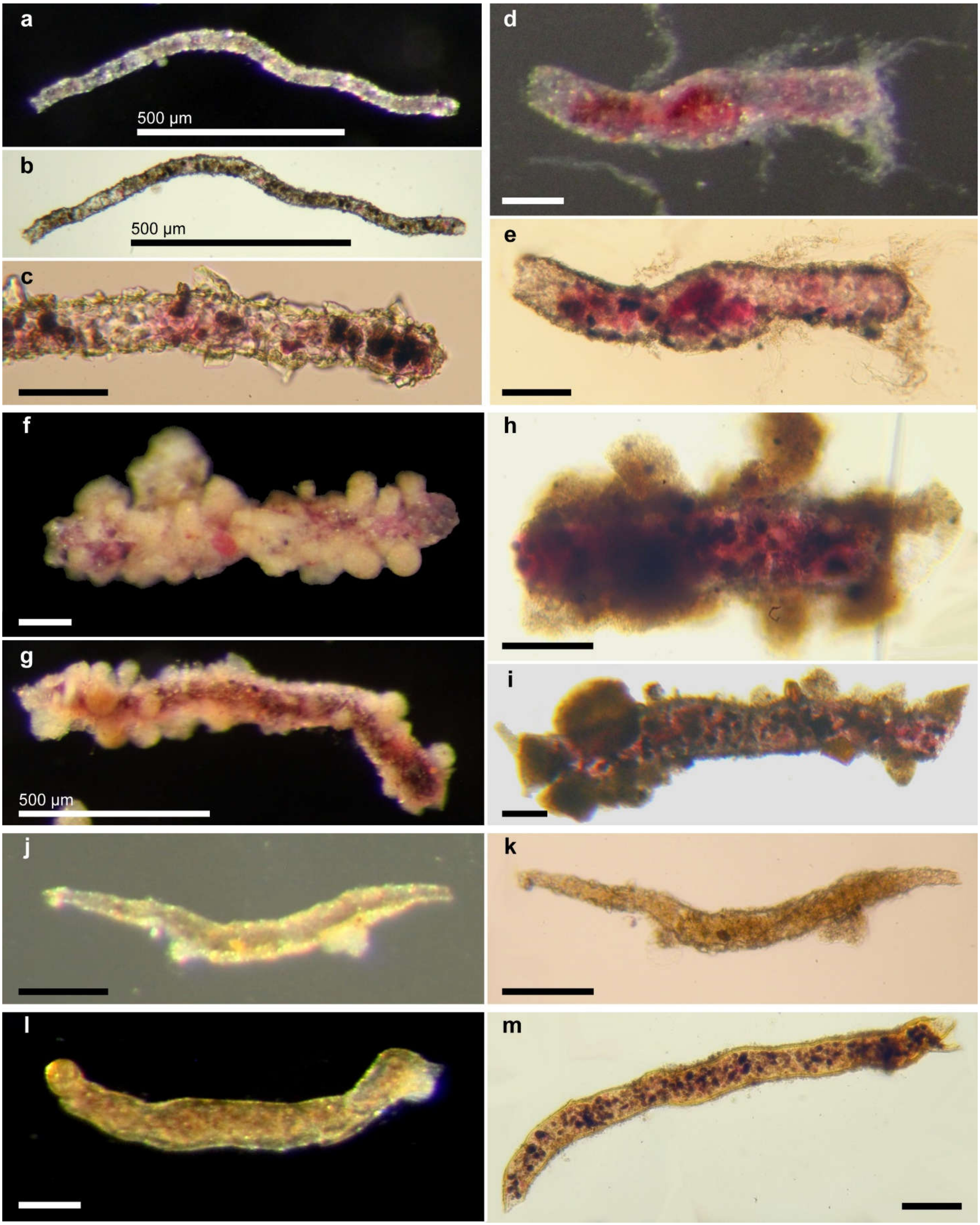

Supplementary Figure S6: Reflected (a, d, i) and transmitted (b, c, e, f–h, j) light photographs of the 4 of the 37 most abundant other monothalamous species represented by complete specimens or fragments (live and dead combined). (a–c) Tube sp. 9; overview of 3 specimens (a) and detail of a fourth specimen (b, c). (d–f) Tube sp. 23; overview (d) and detail (e, f) of same specimen. (g–h) Tube sp. 36 (same specimen). (i–j) Tube sp. 41 (2 specimens). All scale bars 100 µm except where indicated otherwise.

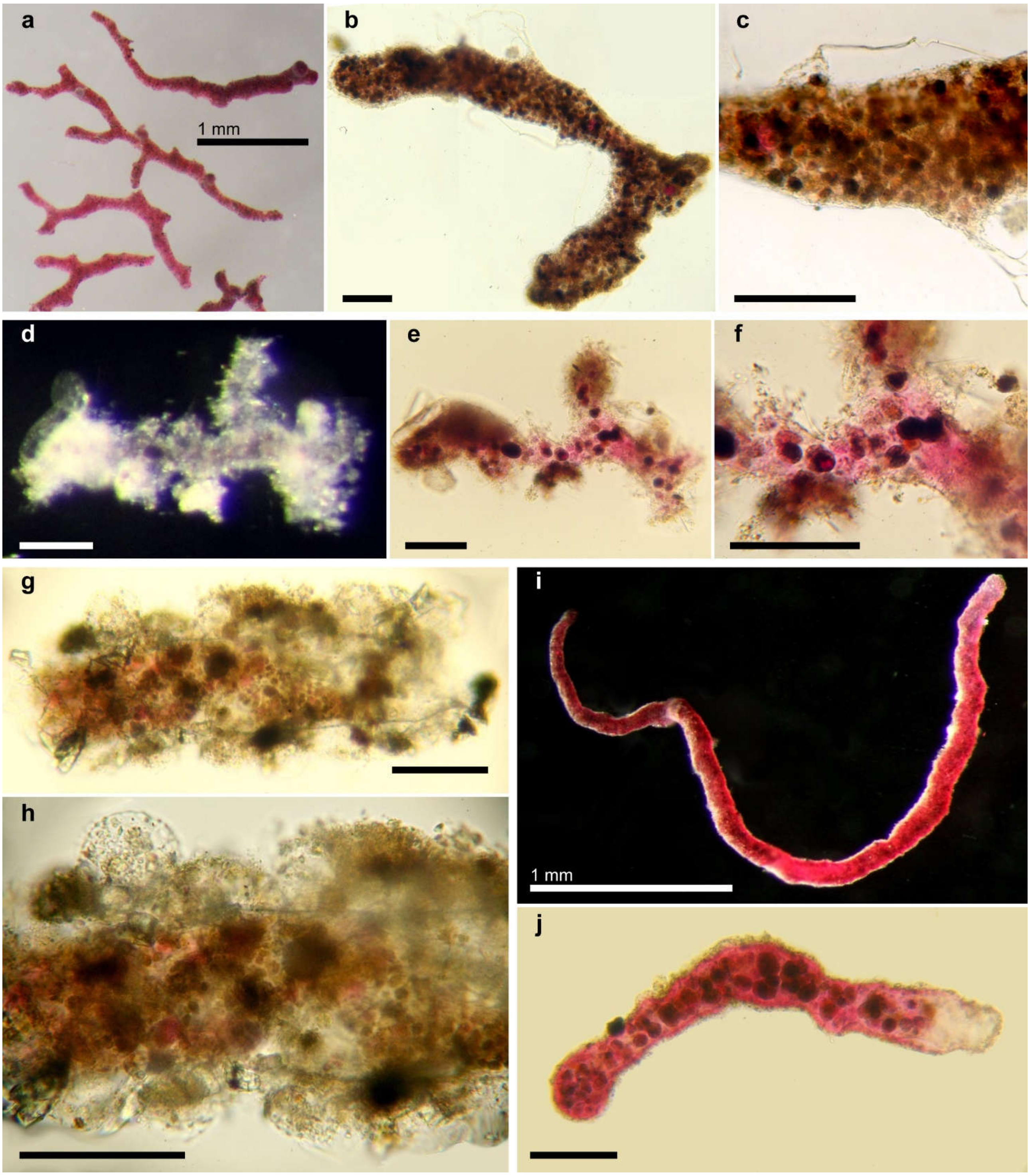

Supplementary Figure S7: Reflected (a, c, e, g, i) and transmitted (b, d, f, h, j) light photographs of 3 of the 37 most abundant other monothalamous species represented by complete specimens or fragments (live and dead combined). (a–f) Tube sp. 45; overview (a, c, e) and detail (b, d, f) of 3 specimens. (g, h) Tube sp. 46 (same specimen). (i, j) Tube sp. 44 (same specimen). All scale bars 100 µm except where indicated otherwise.

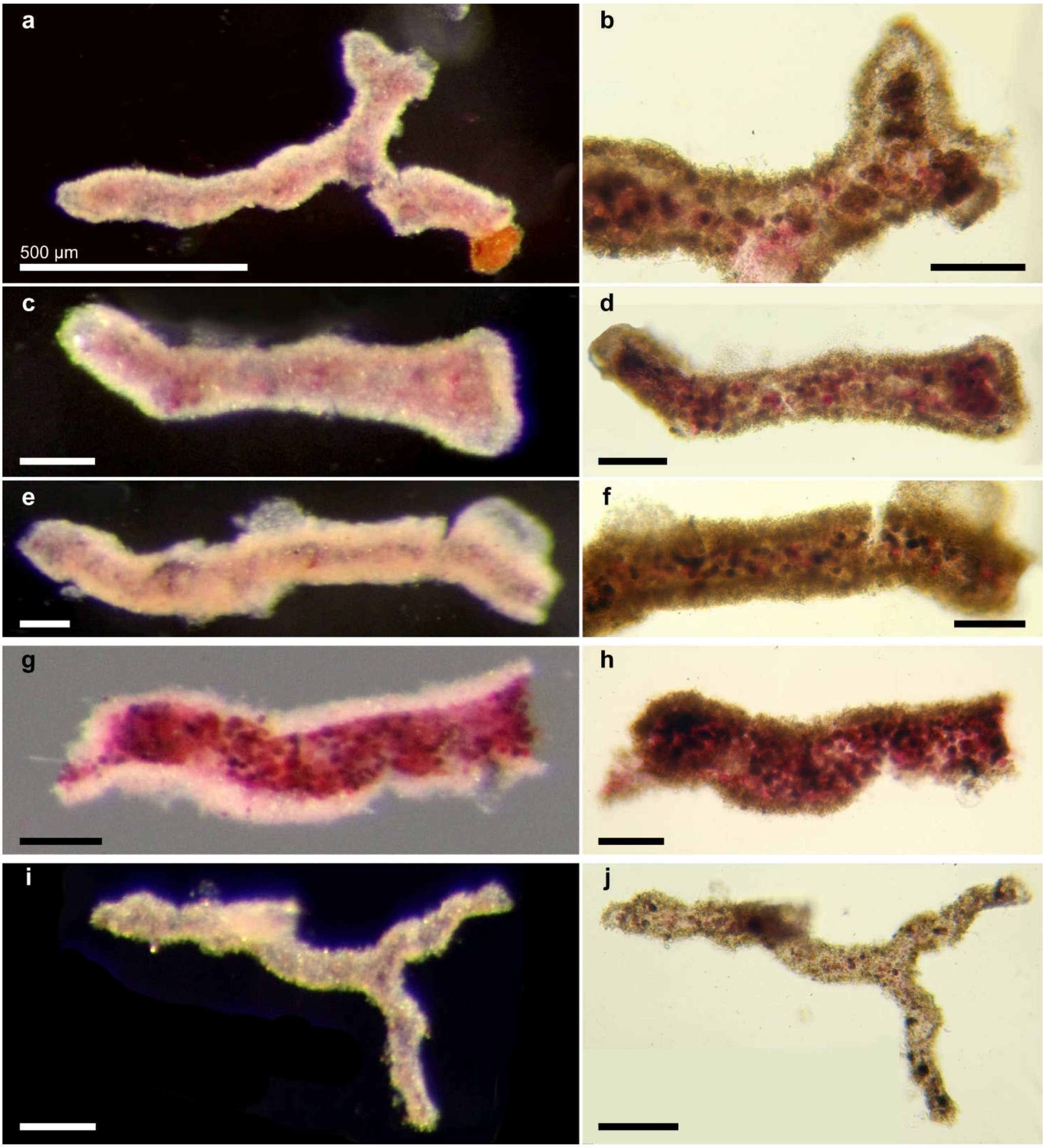

Supplementary Figure S8: Reflected (a, c, e) and transmitted (b, d, f) light photographs of 3 of the 37 most abundant other monothalamous ('pseudo-chambered') species represented by complete specimens or fragments (live and dead combined). (a, b) Chain sp. 3; overview (a) and detail (b) of same specimen. (c, d) Chain sp. 7 (2 specimens). (e, f) Chain sp. 12 (same specimen). All scale bars 100 µm except where indicated otherwise.

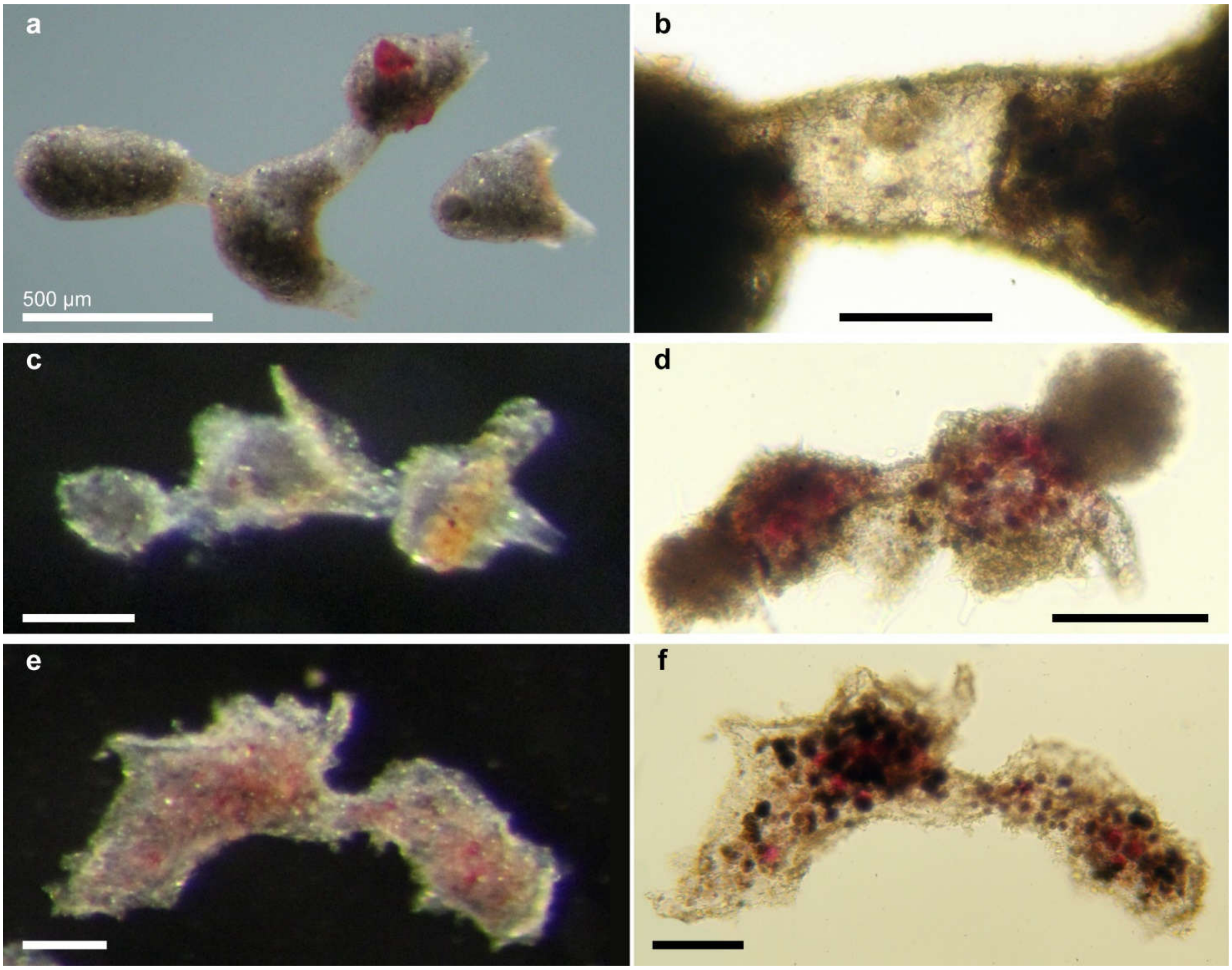

Supplementary Figure S9: Reflected (a, d, g, i) and transmitted (b, c, e, f, h, j) light photographs of 4 of the 37 most abundant other monothalamous species represented by complete specimens or fragments (live and dead combined). (a–c) Monothalamid sp. 17; overview of specimen (a) and details of a second specimen (b, c). (d–f) Monothalamid sp. 19 (2 specimens). (g, h) Monothalamid sp. 23 (same specimen). (i, j) Monothalamid sp. 33 (2 specimens). All scale bars 100 µm except where indicated otherwise.

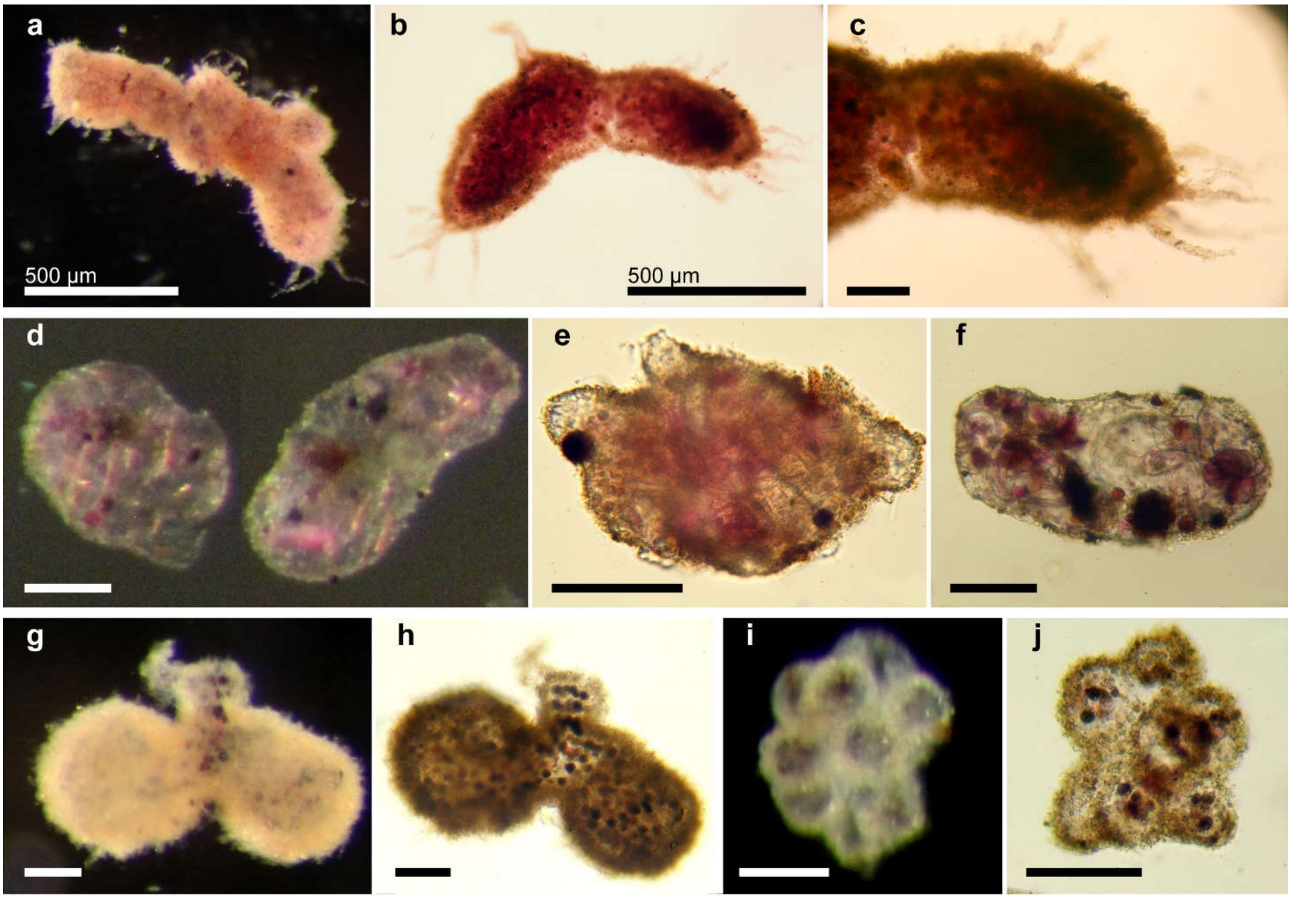

Supplementary Figure S10: Reflected (a, c) and transmitted (b, d) light photographs of 2 of the 5 most abundant species of Baculellidae (komokiaceans) represented by complete specimens or fragments (live and dead combined). (a–b) *Edgertonia* sp. 4; overview of 10 fragments (a) and detail of one individual fragment (b). (c–d) *Edgertonia* sp.5; overview (a) and detail (b) same specimen (d). All scale bars 100 µm except where indicated otherwise.

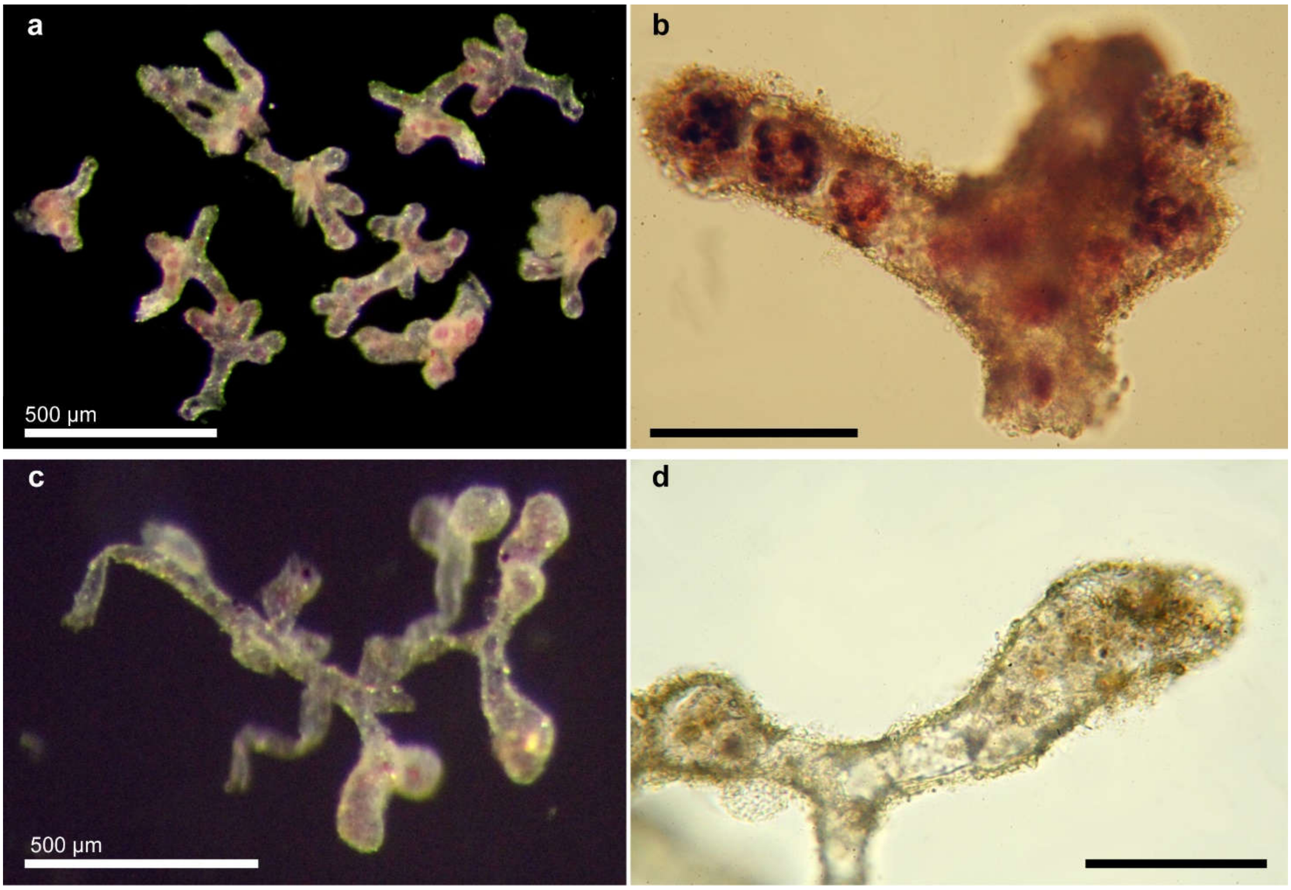

Supplementary Figure S11: Reflected (a, c, e) and transmitted (b, d, f) light photographs of the 3 most abundant species of Komokiidae (komokiaceans) represented by complete specimens or fragments (live and dead combined). (a, b) *Reticulum* sp. 1 (same specimen). (c, d) *Reticulum* sp. 4 (same specimen). (e, f) *Lana* sp. 1 (same specimen). Scale bar 100 µm except where indicated otherwise.

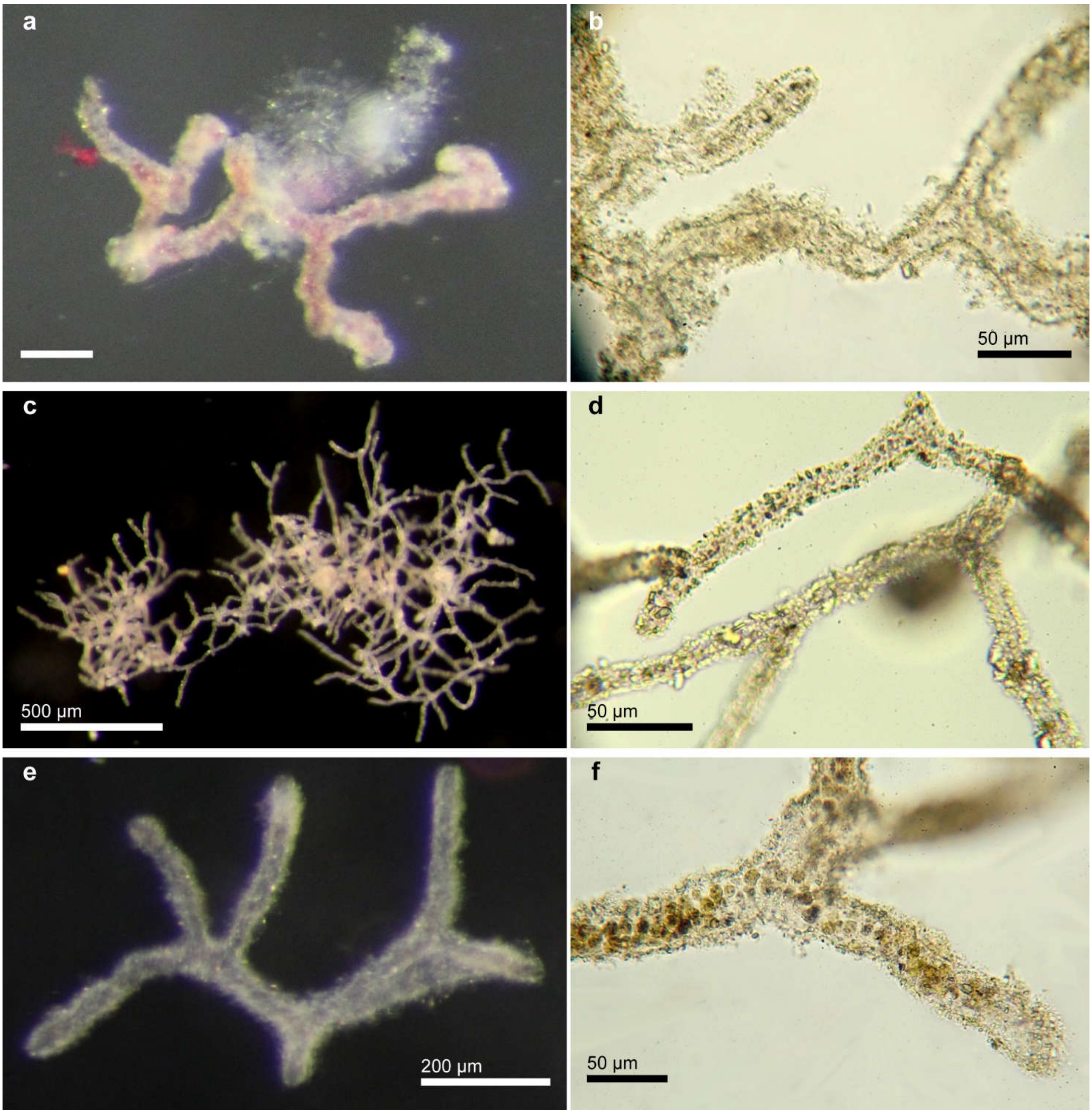

Supplement: Supplementary Information [file srep45288-s1.pdf]
